# Redox signaling modulates axonal microtubule organization and induces a specific phosphorylation signature of microtubule-regulating proteins

**DOI:** 10.1016/j.redox.2025.103626

**Published:** 2025-04-03

**Authors:** Christian Conze, Nataliya I. Trushina, Nanci Monteiro-Abreu, Lisha Singh, Daniel Villar Romero, Eike Wienbeuker, Anna-Sophie Schwarze, Michael Holtmannspötter, Lidia Bakota, Roland Brandt

**Affiliations:** a Department of Neurobiology, School of Biology/Chemistry, Osnabrück University, Germany; b Center for Cellular Nanoanalytics, Osnabrück University, Germany; c Institute of Cognitive Science, Osnabrück University, Germany

**Keywords:** Microtubules, Tau, Axon, Redox signalling, Hydrogen peroxide

## Abstract

Many life processes are regulated by physiological redox signaling, but excessive oxidative stress can damage biomolecules and contribute to disease. Neuronal microtubules are critically involved in axon homeostasis, regulation of axonal transport, and neurodegenerative processes. However, whether and how physiological redox signaling affects axonal microtubules is largely unknown. Using live cell imaging and super-resolution microscopy, we show that subtoxic concentrations of the central redox metabolite hydrogen peroxide increase axonal microtubule dynamics, alter the structure of the axonal microtubule array, and affect the efficiency of axonal transport. We report that the mitochondria-targeting antioxidant SkQ1 and the microtubule stabilizer EpoD abolish the increase in microtubule dynamics. We found that hydrogen peroxide specifically modulates the phosphorylation state of microtubule-regulating proteins, which differs from arsenite as an alternative stress inducer, and induces a largely non-overlapping phosphorylation pattern of MAP1B as a main target. Cell-wide phosphoproteome analysis revealed signaling pathways that are inversely activated by hydrogen peroxide and arsenite. In particular, hydrogen peroxide treatment was associated with kinases that suppress apoptosis and regulate brain metabolism (PRKDC, CK2, PDKs), suggesting that these pathways play a central role in physiological redox signaling and modulation of axonal microtubule organization. The results suggest that the redox metabolite and second messenger hydrogen peroxide induces rapid and local reorganization of the microtubule array in response to mitochondrial activity or as a messenger from neighboring cells by activating specific signaling cascades.

## 1 Introduction

Microtubules are a crucial filament system involved in virtually all aspects of a neuron's life. In the axon, they have a characteristic organization consisting of an array of relatively short microtubules, the plus-end of which is oriented towards the distal tip [1]. Such organization is thought to be critical for efficient axonal transport, in which cargo is carried to the distal axon over long distances. Although axonal microtubules are relatively stable, they still show considerable dynamics, with the plus ends of the microtubules exhibiting stochastic changes between growth and shrinkage, referred to as dynamic instability. Microtubule stability and dynamics are regulated by a variety of microtubule-regulating proteins, including microtubule nucleators, microtubule-binding proteins, end-binding proteins, tubulin-sequestering proteins and microtubule-severing proteins [2]. Further complexity arises from the presence of a variety of different tubulin isoforms. Humans possess eight α− and nine β−tubulin genes, most of which are expressed in neurons [3], as well as a multitude of different posttranslational modifications (PTMs) of the tubulin subunits, referred to as the tubulin code [4]. Also many microtubule-regulating proteins exhibit PTMs that modulate their activity. The best studied of these is phosphorylation, and increased phosphorylation of specific serine and threonine residues is known to influence the interaction of neuronal microtubule-associated proteins (MAPs) such as MAP1B and tau, with microtubules. Of disease-related importance is the modulation of the interaction of the MAP tau with axonal microtubules, as tau is hyperphosphorylated in Alzheimer's disease and other tauopathies, which largely reduces its interaction with microtubules [5].

Oxidative stress is associated with many neurodegenerative diseases, and increased levels of reactive oxygen species (ROS) may play a role in triggering axon degeneration as well as microtubule disorganization and disassembly [2,6]. High levels of ROS form additional free radical compounds that have the potential to damage lipids, proteins, and nucleic acids over time [7], inhibit axonal transport [8] and induce axonal degeneration, as evidenced by the morphological features of axon beading and fragmentation [9]. However, more recently it has become clear that moderately elevated ROS levels can act as a crucial physiological mediator of many cellular processes and regulator of intrinsic signaling pathways [10,11].

The most common ROS members in mediating oxidative eustress are the superoxide anion, the hydroxyl radical, and hydrogen peroxide. Because these ROS species act as relatively short-lived second messengers, they are able to regulate intrinsic signaling pathways [11]. Hydrogen peroxide was found to be the most important redox metabolite involved in signal transduction and redox regulation. It is an electrically neutral molecule and is chemically more stable than the other members of the ROS family. As a messenger molecule, hydrogen peroxide diffuses or distributes through cells and tissues by passive transport to trigger immediate cellular effects that link redox biology with regulation through phosphorylation and dephosphorylation [12]. Physiological hydrogen peroxide levels promote the establishment of neuronal polarity, neurite growth and axon specification [13]. While all of these processes critically depend on the regulation of microtubule polymerization, the mechanisms of how hydrogen peroxide influences axonal microtubules and microtubule-dependent functions are largely unknown.

Here, we determined the effect of subtoxic concentrations of hydrogen peroxide on axonal microtubule dynamics using quantitative live-cell imaging. We analyzed changes in the structure of the axonal microtubule array using single-molecule localization microscopy and algorithm-based reconstruction of microtubules. We examined possible functional microtubule-dependent consequences by tracking transport vesicles and determining the tau-microtubule interaction using fluorescence-decay after photoactivation (FDAP) experiments. To identify downstream targets, we performed proteomic and phosphoproteomic analysis of differentiated neurons treated with subtoxic hydrogen peroxide and compared it with arsenite as an alternative stressor.

## 2 Materials and methods

### 2.1 Materials and animals

Unless otherwise stated, chemicals and cell culture material were purchased from Sarstedt (Nümbrecht, Germany), Sigma-Aldrich (Deisenhofen, Germany), and Thermo-Fisher Scientific (Waltham, USA). The microtubule-targeting agent epothilone D (EpoD) was a kind gift from Amos Smith 3rd (University of Pennsylvania) and was prepared as previously described [14,15]. The purity of the compound was >95 %, as determined by LC-MS and NMR analyses. The spectroscopic properties were identical to those reported in the literature. The mitochondria-targeted antioxidant SkQ1, and TPP, which lacks the antioxidant quinone moiety, were provided by Maxim Skulachev (Mitotech S.A., Luxembourg).

Mice were kept and killed in accordance with the German animal care regulations based on the FELASA guidelines. One year old C57Bl/6J mice (Envigo, Netherlands) were used to prepare neurons from the dorsal root ganglia (DRG).

### 2.2 Cell culture and transfections

PC12 cells were cultured in serum-DMEM and transfections were performed with Lipofectamine 2000 (Thermo-Fisher Scientific, USA) as previously described [16]. Expression plasmids for PAGFP-α-Tubulin and mEGFP-α-Tubulin have been described previously [17]. The expression plasmid PAGFP-htau441wt [18] was used to express the longest CNS-isoform of tau. pEGFP-n1-APP was obtained from Zita Balklava and Thomas Wassmer (Addgene plasmid #69924; http://n2t.net/addgene:69924; RRID: Addgene_69924).

DRGs were isolated under sterile conditions from adult 1-year-old male C57BL/6J mice. After dissection, DRGs were collected in ice-cold Hank's Balanced Salt Solution (HBSS) supplemented with 1 % penicillin/streptomycin, 15 mM glucose, and 5 mM HEPES. Ganglia were then enzymatically digested with papain and collagenase/dispase at 37 °C to facilitate dissociation. After digestion, ganglia were triturated with a fire-polished Pasteur pipette in NB medium (Neurobasal with 2 % B27 supplement, 1 % glutamine, 1 % fetal bovine serum, 1 % horse serum, 25 μM beta-mercaptoethanol, and 100 μg/ml primocin), to obtain a single-cell suspension. The dissociated neurons were collected by centrifugation and plated on Matek dishes coated with poly-l-lysine and laminin. Cells were maintained at 37 °C in NB medium containing 50 ng/ml 7S mouse NGF in a humidified incubator with 5 % CO_2_.

### 2.3 Metabolic activity and cytotoxicity profiling using a combined LDH and MTT assay

PC12 cells were cultured in 96-well plates at 1 × 10^4^ cells/well in serum-reduced medium supplemented with 100 ng/ml 7S mouse NGF. Cells were incubated for 48 h to initiate neuronal differentiation. Concentration-response profiling of hydrogen peroxide (H_2_O_2_) was performed with four triplicates and six concentrations ranging from 50 to 900 μM. The respective H_2_O_2_ concentration was added to each well 3 h before the measurement. Designated wells for controls were untreated for negative control or a total cell lysis was induced by the addition of 1 % Triton X-100 for a positive control. For the LDH assay, 50 μl medium from each well of the assay plate was transferred to a separate 96-well plate. For quantification of the LDH release, 50 μl of LDH reagent (4 mM iodonitrotetrazolium chloride (INT), 6.4 mM beta-nicotinamide adenine dinucleotide sodium salt (NAD), 320 mM lithium lactate, 150 mM of 1-methoxyphenazine methosulfate (MPMS) in 0.2 M Tris-HCl buffer, pH 8.2) was added. The plate was shaken for 10 s and incubated in the dark for several minutes. Absorbance was measured at 490 nm using a Thermomax Microplate Reader operated with SoftMaxPro Version 1.1 (Molecular Devices Corp., Sunnyvale, CA, U.S.A.). LDH release measurements were normalized to the optical densities of the positive control wells. For the MTT assay, MTT reagent (3,(4,5.dimethylthiazol-2-yl)2,5-diphenyltetrazolium bromide) at a final concentration of 1 mg/ml MTT was added to the wells in the remaining assay plate. The cells were incubated for a further 2 h in the cell culture incubator before the reaction was stopped by adding 50 μl of lysis buffer (20 % (wt/vol) sodium dodecyl sulfate in 1:1 (vol/vol) N,N-dimethylformamide/water, pH 4.7). After overnight incubation at 37 °C, the optical densities of the formazan product were determined at 570 nm. MTT conversion measurements were normalized to the optical densities of the negative control wells.

### 2.4 Detection of intracellular ROS levels

Intracellular ROS levels were assessed using the Cellular ROS Assay Kit Orange (abcam, ab186028, Cambridge, UK). PC12 cells (2 × 10^4^ cells/well) were seeded in 60 μl of serum-reduced DMEM supplemented with 100 ng/ml 7S mouse NGF in a 96-well plate. To measure the effect of SkQ1, 30 μl of NGF containing serum-reduced medium supplemented with 0.2 μM SkQ1 or carrier control (0.2 % EtOH) were added after 24 h. After a further 24 h of incubation, the cells were treated with the respective H_2_O_2_ concentration (150 μM and 450 μM) for 3 h. A total of six independent experiments were conducted. Intracellular ROS levels were determined according to the manufacturer's instructions. Briefly, cells were incubated with 100 μl of ROS Orange Working solution for 60 min. Changes in fluorescence intensity were measured with the FLUOstar Optima (BMG Labtechnologies, Ortenberg, Germany) at Ex/Em = 540/570 nm. The fluorescence intensities were normalized to the respective negative control values after background subtraction.

### 2.5 Live-cell imaging

Fluorescence decay after photoactivation (FDAP) experiments with PAGFP-α-tubulin- or PAGFP-tau-expressing cells were performed essentially as described previously [19]. Briefly, PC12 cells were plated on 35-mm poly-l-lysine and collagen-coated glass-bottom culture dishes (MatTek, USA). After transfection, PC12 cells were neuronally differentiated by media exchange with DMEM with 1 % (vol/vol) serum containing 100 ng/ml 7S mouse NGF (Alomone Laboratories, Germany). Cultivation was continued for 4 days with medium exchange to DMEM with 1 % (vol/vol) serum containing NGF and without phenol red one day prior to live imaging. Live imaging of PC12 cells for photoactivation experiments was performed using a Nikon Eclipse Ti2-E laser scanning microscope (Nikon, Japan) equipped with a LU-N4 laser unit with 488-nm and 405-nm lasers, a Fluor 60 × ultraviolet-corrected objective lens (NA 1.4), and a C2+ scanner enclosed in an incubation chamber at 37 °C and 5 % CO_2_. Automated image acquisition of PAGFP-tubulin- or PAGFP-tau-expressing cells after photoactivation was performed essentially as described previously [20]. Briefly, photoactivation of a 6 μm long neurite segment was performed with a 405-nm laser. A set of consecutive image series (time stacks) was created at a frequency of 1 frame/s and 112 frames were collected per activated cell with a resolution of 256 × 256 pixels.

Live-cell imaging to analyze APP transport in PC12 cells was performed essentially as previously described [21] using a Zeiss Cell Observer Z1 (Zeiss, Germany) with high-speed confocal imaging using the CSU-X1 spinning disc technology from Yokogawa, equipped with an optically pumped 488-nm semiconductor laser surrounded by a sample incubation chamber controlled by a Zeiss TempModule S1. pH stability was maintained by adding 30 mM HEPES buffer to the cell culture medium before image acquisition. For image capture of eGFP-APP vesicle tracking, the 488-nm laser and Alpha Plan-Apochromat × 63 (NA 1.46) objective were used. An image series (time stack) of 60 s was acquired at a speed of five images per second, captured with a Hamamatsu ORCA flash V3 and 2 × 2 binning at a resolution of 640 × 640 pixels (pixel size 172 nm). The image series were deconvolved with the software Huygens Remote Manager v3.5 (Scientific Volume Imaging B.V., Hilversum, Netherlands) using a theoretical PSF and a classical maximum likelihood estimation as the deconvolution algorithm with 20 iterations and a quality criterion of 0.05.

Treatment of PC12 cells with 150 μM H_2_O_2_ was performed 3 h before live imaging. To assess the effect of the antioxidant SkQ1 in FDAP experiments, cells were treated with 0.2 μM of the compound 24 h before H_2_O_2_ addition. To determine the effect of H_2_O_2_ treatment on stabilized MTs by epothilone D (EpoD) in FDAP experiments as well as APP vesicle tracking, cells were treated with 1 nM EpoD or its carrier control (0.01 % DMSO) 1 h before H_2_O_2_ treatment.

### 2.6 FDAP data analysis

A reaction-diffusion model was used to determine the association rate k∗_on_ and the dissociation rate k_off_ constant of tubulin or tau binding, as described previously [20]. Processing and analysis of individual FDAP curves was performed as previously described [19] using a custom C-based tool called cFDAP. The fitting procedure was used to obtain k∗_on_ and k_off_ from each individual FDAP curve, while the χ2 value was used as an indicator of the goodness of fit of the model function. Effective diffusion constants were obtained by fitting fluorescence decay data from photoactivation experiments using a one-dimensional diffusion model function for FDAP, as previously described [22].

### 2.7 Morphometric analysis

For morphometric analysis, 5 × 10^4^ PC12 cells were seeded per poly-l-lysine and collagen-coated 12 mm coverslips in 4-well plates and differentiated with NGF for 4 days as described above. Cells were then treated with 150 μM H_2_O_2_ for 3 h or left untreated for the same time and processed with a combined NP-40 permeabilization-fixation protocol [23]. For immunostaining, monoclonal antibody against β-tubulin (AA10, Invitrogen, USA) and Alexa Fluor 488-conjugated goat anti-mouse IgG (H + L, Superclonal Recombinant Secondary Antibody, Invitrogen, USA) were used. Images of representative microscopic fields were acquired using a 40 × oil objective with a 1024 × 1024 pixel resolution using a Nikon Eclipse Ti2-E laser scanning microscope (Nikon, Japan) with a 488-nm laser. Analysis was performed using Fiji (Image J 1.54k). Cell body area was measured using the elliptical tool, followed by measuring the diameter using the segmented line tool. The length of cell processes was measured using the segmented line tool; only processes that exceeded the radius of the cell body were considered as processes. The measured values were exported to Excel spreadsheets for further analysis. For each model neuron, the number of processes, the diameter of the cell body, the radius of the cell body, the length of each process, the total process length (calculated as the sum of all process lengths for each cell), and the mean process length (calculated by dividing the total process length by the number of processes per cell) were determined.

### 2.8 Tracking and quantifying vesicle transport

Transport of APP-vesicles was tracked and analyzed using Imaris v 9.2 (Bitplane, Oxford Instruments). Vesicles were detected by the Gaussian-filtered intensity of their signal within a diameter of 500 nm around the respective signal peak. Vesicle transport was tracked by an autoregressive motion algorithm with a maximum distance of a future position of the signal of 1 μm and a maximum gap size of 2 frames (400 ms). To determine the direction of the tracked vesicles, a reference point at the transition from the cell body to the neurite was used, while only the tracks that spanned a period of at least 3 s (15 frames) were considered for further analysis. Quantified transport parameters were exported to spreadsheets (Excel, Microsoft Corporation, USA) and further processed using TIBCO Spotfire Data Analysis Software (TIBCO Software Inc., USA). Vesicles that exceeded a displacement of more than 0.75 μm over the observation time of 60 s were designated as mobile, otherwise as stationary. To determine the proportion of mobile versus stationary vesicles, between 150 and 220 tracks per cell were evaluated.

### 2.9 Super-resolution microscopy with DNA-PAINT

For super-resolution microscopy, PC12 cells were transfected with mEGFP-α-Tubulin and neuronally differentiated for 4 days as described above. Treatment with 150 μM H_2_O_2_ was performed 3 h before fixation or was omitted as control. Fixation, immunostaining, and super-resolution microscopy with DNA-PAINT were performed essentially as previously described [17]. Briefly, PC12 cells were processed using a combined NP-40 permeabilization-fixation protocol, which removes membranes and cytosolic components but preserves cytoskeletal structures and associated proteins [24]. Immunostaining used an anti-GFP nanobody obtained from the Massive-Tag-Q Anti-GFP DNA-PAINT kit (Massive Photonics GmbH, Gräfelfing, Germany). Just before imaging, cells were washed three times with PBS and incubated with a 1:1000 dilution of 50 nm gold nanorods (Nanopartz, E12-50-600-25) that function as fiducial markers during image acquisition. Afterwards, cells were washed three times with PBS before an imaging buffer (Massive Photonics) supplemented with 250 pM Cy3b-conjugated imager DNA-strand, complementary to the DNA strand attached to the anti-GFP nanobody, was added. Cells were imaged by total internal reflection fluorescence (TIRF) microscopy in the highly inclined and laminated optical sheet (HILO) mode [25] using an inverted microscope frame (Olympus IX-81) equipped with a motorized quad-line TIR illumination condenser (cellTIRF-4-Line, Olympus) and a motorized xy-stage (Märzhäuser Scan IM 120 × 80). Three-dimensional single-molecule localization was achieved by astigmatic imaging using a cylindrical lens (Olympus) implemented directly in front of the filter wheel.

Super-resolution microscopy of DRG neurons was performed in essentially the same manner, except that immunostaining was performed after NP-40 permeabilization-fixation with a monoclonal anti-β-tubulin antibody (AA10, Invitrogen, USA) and Alexa Fluor 488-conjugated goat anti-mouse IgG (H + L, Superclonal Recombinant Secondary Antibody, Invitrogen, USA) to visualize and select suitable neurons for imaging for DNA-PAINT. For DNA-PAINT of the identified cells, microtubules were first stained with a polyclonal anti-α-tubulin antibody (PA5-19489; Invitrogen, USA), followed by the application of an oligonucleotide-labelled anti-rabbit nanobody from Massive-SDAB 1-PLEX kit (Massive Photonics GmbH, Gräfelfing, Germany). To visualize the microtubule array, the imager from the rabbit-specific Massive-SDAB 1-PLEX kit was used.

### 2.10 Post-processing of DNA-PAINT datasets

Raw data sets were processed using the "Picasso" software package [26], https://github.com/jungmannlab/picasso). At the beginning of each imaging session, a z-stack of immobilized fluorescent TetraSpeck™ microspheres with a diameter of 100 nm (Invitrogen, T7279) was acquired. This was done in the respective imaging buffer with a step size of 10 nm using a piezo z-stage (NanoScanZ, NZ100, Prior Scientific). This is a necessity for the calibration of astigmatic PSFs, which is a prerequisite for three-dimensional single-molecule localization microscopy. This z-stack was analyzed using “Picasso: Localize” with parameters, that allow the identification of single beads in each frame. The photon conversion parameters were set as follows: EM Gain: 1, Baseline: 400, Sensitivity 0.46, Quantum Efficiency: 0.80 and pixel size: 130 nm. A calibration file was generated with the "Calibrate 3D" function of "Picasso localize". This file, as well as the same photon conversion parameters, were used for image processing of raw sample files. The Min. Net. Gradient was adjusted to remove non-specifically bound imager strands or other background signals to filter for localizations with the highest signal intensities. Single-molecule localizations were fitted with a Gaussian least-square fit. For 3D localization, the magnification factor was set to 1.0. Processed data sets were opened with "Picasso: Render" and a drift correction with cross-correlation was performed, followed by a correction using the fiducials. The localizations of these datasets were then exported for the ImageJ plugin ThunderSTORM for 3D rendering to obtain image-stacks with a well-defined voxel size set to 26 nm × 26 nm × 25 nm based on the overall axial resolution of 25 nm [27].

### 2.11 SIFNE analysis of post-processed DNA-PAINT data

Computational analysis and quantification of the microtubule array in axon-like processes of PC12 cells was performed using the open-source SIFNE (SMLM image filament extractor) software package [28]. The MATLAB-based tool involves the iterative extraction of the filamentous structures from the image data set and the subsequent identification and assignment of the detected filaments. Since the axial distance between microtubules in PC12 neurites is about 70 nm [29], in the case of PC12 cells, we chose optical sections with a thickness of 150 nm for filament extraction by SIFNE. Considering that axons of DRG neurons are structurally thinner than processes of PC12 cells, we used optical sections with a thickness of 70 nm in DRG neurons for filament extraction by SIFNE. This allowed us to use sections where the resolution was highest while avoiding overlapping microtubules from another plane that would confound our statistics. The region of interest (ROI) in axon-like processes of PC12 cells was set to the shaft of the neurite. For DRG neurons, for image enhancement using line and orientation filter transformation algorithms (LFT and OFT), a radius of 10 pixels with 40 rotations of the scan line segment was used. Segmentation was performed using SIFNE's automatic thresholding function based on Otsu's method. For creating a pool of minimal linear filament fragments, areas of filament connections were removed by a local area around each connection of 2 × 2 pixels. To recover unrecognized linear structures, iterative extraction of the filaments was performed twice, followed by registration of the propagation direction of each filament tip. The grouping and analysis of the detected filaments was carried out with a pixel size of 26 nm and a maximum curvature of 1 rad/μm. The search angle and radius were set to 60° and 40 pixels, respectively. The allowable orientation difference between the endpoints was set to 60°. The maximum allowable angle difference and endpoint gap vector were set to 60° and 30°, respectively. The weights for similarity and continuity conditions during the scoring calculations were set to 1. Due to the high complexity of the cytoskeletal network, fragment overlap was not allowed as suggested by Ref. [28]. For sorting composite filaments, the minimum filament length was set to 15 pixels corresponding to 390 nm, while ungrouped filaments were left in the data set.

### 2.12 Proteome and phosphoproteome analysis

PC12 cells were neuronally differentiated by culture in serum-reduced DMEM with 100 ng/ml 7 S mouse NGF for 4 days and treated with 150 μM H_2_O_2_ for 3 h, 0.5 mM arsenite for 20 min, or left as respective controls. Proteome and phosphoproteome analysis were essentially performed as described previously [30]. Briefly, cells were incubated in lysis buffer (8 M urea in 50 mM Tris/HCl, pH 7.8) supplemented with Phos-Stop tablets (Roche Diagnostics GmbH, Germany), sonicated and cleared by centrifugation. Protein concentrations were determined using Pierce™ BCA Protein Assay (Thermo Fisher Scientific, USA). 0.2 μg/μl α-casein was added to a protein amount of 1.2 mg, and reduction and alkylation were carried out in lysis buffer containing 15 mM iodoacetamide and 5 mM DL-dithiothreitol. The proteins were digested with trypsin/Lys-C Mix (Promega Corporation, USA), and 10 μg of each sample was used for proteomic analysis. For phosphoenrichment, the remaining samples were desalted using Sep-Pak® Classic C18 cartridges (Waters, Ireland). The eluate was lyophilized and enriched with a High-Select™ TiO_2_ Phosphopeptide Enrichment Kit (Thermo Fisher Scientific, USA). For proteome and phosphoproteome analysis, samples were collected from a PepMap C18 easy spray column (Thermo Fisher Scientific, USA). MS analysis was performed as previously described [31]. The ∗.raw data files were analyzed using PEAKS Online software (Bioinformatic Solutions Inc, Canada). PEAKS Q (de-novo-assisted quantification) analysis was used for data refinement with mass correction, de novo sequencing and de novo-assisted database search, and subsequent label-free quantification. The search engine was applied to *Rattus norvegicus* ∗.fasta databases. MS/MS searches were performed using a mass tolerance of 10 ppm parent ions and a mass tolerance of 0.2 Da fragments. Trypsin with up to two missing cleavage sites was selected as the cleavage enzyme. The carbamidomethylation modification was chosen as the fixed modification and the oxidation of methionine, the acetylation of lysine and the phosphorylation of serine, threonine and tyrosine as variable modifications. A maximum of three variable modifications were allowed per peptide. Normalization to the total ion current level was performed for each sample. The ANOVA test was used to calculate the significance level for each protein, outliers were removed, and the top three peptides were used to quantify the protein signal where possible. The peptide identification was considered valid at a false detection rate of 1 % (q-value <0.001) (maximum delta Cn of the percolator was 0.05). The minimum length of acceptable identified peptides was set to six amino acids. Each condition was analyzed in triplicate. All proteins were assigned their gene symbol via the Uniprot knowledge database (http://www.uniprot.org/).

### 2.13 Bioinformatic analysis

Tubulin and microtubule-regulating proteins highlighted on the volcano plots were based on the classification of different functional groups [32]: tubulin isoforms (α- and β-tubulins), nucleators (γ-tubulins and γ-tubulin complex proteins (GCPs)), MT-binding proteins (MAP1A, MAP1B, MAP1S, MAP2, tau (encoded by the MAPT gene), MAP4, MAP6 (STOP) and MAP7 (ensconsin)), tubulin-sequestering proteins (stathmins, encoded by STMN1, STMN2 (SCG10), STMN3 (SCLIP), STMN4 (RB3)), end-binding proteins (EB1, EB2, EB3 (encoded by MAPRE1, MAPRE2, MAPRE3, respectively), CLASP1 and CLASP2), MT-severing proteins (P60-katanin (encoded by KATNA1 and KATNB1), fidgetin (FIGN) and spastin (SPAST)). Gene Ontology (GO) term enrichment analysis was performed to identify overrepresented Molecular Function (MF) and Cellular Component (CC) categories using EnrichR [33]. All differentially expressed proteins and all proteins with upregulated phosphosites were used to create gene sets for enrichment analysis. The 10 highest-scoring GO-terms for molecular function and cellular component were displayed. Gene Ontology (GO) regarding molecular function and cellular component was used to construct of protein classifications. Kinase enrichment analysis (KEA) was performed using the curated kinase-substrate database KEA2 [34]. The gene symbols with phosphorylated sites of differentially phosphorylated proteins were used to infer upstream kinases whose putative substrates are overrepresented. To identify the most important functional pathways upon treatment with hydrogen peroxide or arsenite, significantly enriched kinases were compared. As a proxy to predict phosphatase activity, we used the DTL-DephosSite model [35], a deep learning tool for predicting protein dephosphorylation sites. It uses transfer learning based on the prediction of kinase-specific phosphorylation sites and captures common sequence patterns and biochemical features between phosphorylation and dephosphorylation. Peptides that showed significantly lower phosphorylation under the experimental conditions compared to the control were extracted in a 31-residue window with 15 residues before and 15 residues after the phosphorylation site in question. Specific phosphatases were predicted using DEPOD (human DEPhOsphorylation database; http://depod.bioss.uni-freiburg.de/). The human orthologues were translated into the rat orthologues and the number of peptides was plotted against the respective phosphatase.

### 2.14 Statistical analysis

Statistical analysis was performed using GraphPad Prism v8.0.1 (GraphPad Software, USA). All datasets were tested for normality using the D'Agostino-Pearson and Shapiro-Wilk tests. When necessary, datasets were log transformed to allow further statistical testing. Statistical outliers were identified using the ROUT method. Homogeneity was assessed using the Levene test. An unpaired two-tailed *t*-test was used to compare two datasets. In cases of unequal variances, Welch's correction was applied. To compare more than two data sets, one-way ANOVA was performed followed by Dunnet post-hoc test. All statistical values are expressed as mean ± SEM.

## 3 Results

### 3.1 Subtoxic concentrations of hydrogen peroxide decrease microtubule polymer in axon-like processes and increase microtubule dynamics

The redox metabolite hydrogen peroxide is known to act as a messenger molecule and diffuses or distributes through cells and tissues by passive transport [12,36]. While high concentrations or impaired compartmentalization of ROS species are considered toxic (“oxidative distress”) [37], low levels of hydrogen peroxide have been shown to promote neuronal development and axon specification [13], thus positively modulating cellular functions through a process called “oxidative eustress” [11]. Therefore, we first determined the level of subtoxic concentrations of hydrogen peroxide in model neurons, which are likely to induce eustress mechanisms in the cells.

**Fig. 1:**
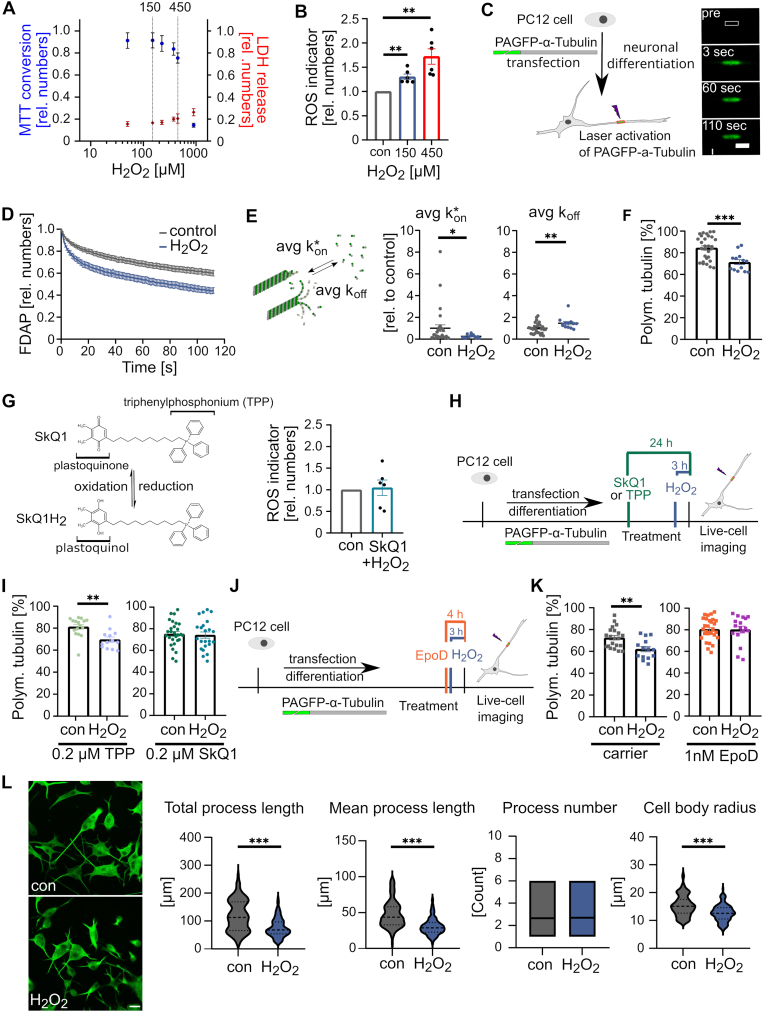
Subtoxic concentrations of hydrogen peroxide decrease microtubule polymer in axon-like processes and increase their dynamics. **A.** Combined MTT (blue) and LDH (red) assay showing the effect of 3 h H_2_O_2_ exposure on neuronally differentiated PC12 cells. Mean ± SEM of 4 experiments, each carried out in triplicates, are shown. Statistically significant differences as determined by one-way ANOVA followed by a Dunnett post hoc test showed significant differences from a control at concentrations ≥225 μM. **B.** Bar graphs showing the increase in intracellular ROS level in response to H_2_O_2_. Each data point represents an independent experiment normalized to a control. Mean ± SEM is shown. Statistically significant differences between treated and control cells as determined by one-sample t-tests are indicated (∗∗p < 0.01). **C.** Representative time-lapse images of a fluorescence decay after photoactivation (FDAP) experiment in an axon-like process. A 6 μm long segment (white box) in the middle of a process was photoactivated and the fluorescence decay in this area was monitored over time. A schematic representation of the FDAP approach and the expressed construct is shown on the left. **D.** FDAP diagrams after photoactivation of PAGFP-α-Tubulin expressing cells show an increased fluorescence decay after treatment with H_2_O_2_. Mean values ± SEM of 29 (control) and 13 (150 μM H_2_O_2_) cells are shown. **E.** Scatterplots of the association (k∗_on_) and dissociation rate constants (k_off_), determined by modelling the FDAP plots from (D), show that H_2_O_2_ decreases k∗_on_ and increases k_off_. Statistically significant differences between treated and control cells, determined by unpaired two-tailed Student's t-tests, are indicated. ∗p < 0.05, ∗∗p < 0.01. **F.** The scatterplot of the amount of polymerized tubulin determined from the association and dissociation constants in (E) shows that H_2_O_2_ reduces the amount of polymerized tubulin in axon-like processes. Statistically significant differences determined by unpaired two-tailed Student's t-tests are indicated. ∗∗∗p < 0.001. **G.** A schematic representation of the conversion between the oxidized and reduced forms of SkQ1 as a mitochondria-targeted antioxidant is shown. TPP, which lacks the antioxidant quinone moiety, is indicated. A bar graph is displayed on the right showing that pretreatment with SkQ1 abolished the increase in intracellular ROS levels in response to H_2_O_2_. **H.** Schematic representation of the timeline of the experiment to determine the effect of pretreatment with SkQ1 or a control (TPP) on microtubule polymerization. **I.** Scatterplot of the amount of polymerized tubulin with the control (TPP) and SkQ1, showing that pretreatment with SkQ1 prevents H_2_O_2_-induced microtubule depolymerization in axon-like processes as determined by FDAP experiments. Shown are mean values ± SEM of 16, 13 (TPP) and 28, 23 (SkQ1) cells for control and H_2_O_2_-treatment, respectively. Statistically significant differences determined by unpaired two-tailed Student's t-tests are indicated. ∗∗p < 0.01. **J.** Shown is the time course of the experiment to determine the effect of pretreatment with the microtubule-stabilizer EpoD on H_2_O_2_-induced microtubule depolymerization. **K.** Scatterplot of the amount of polymerized tubulin showing that pretreatment with EpoD prevents H_2_O_2_-induced microtubule depolymerization. Shown are mean values ± SEM of 21, 14 (carrier, 0.01 % DMSO) and 29, 17 (1 nM EpoD) cells for control and H_2_O_2_-treatment, respectively. Statistically significant differences determined by unpaired two-tailed Student's t-tests are indicated. ∗∗p < 0.01. **L.** Morphometric analysis of the effect of subtoxic H_2_O_2_ concentrations on neuronally differentiated PC12 cells, showing that H_2_O_2_ reduces process length and cell body area. Fluorescence micrographs of representative cells fixed and stained against β-tubulin using a fixation extraction protocol are shown on the left. Violin plots (with mean and standard deviation) and bar graphs (with mean) show total and mean process length, mean process number per cell, and cell body radius. Cells were treated with H_2_O_2_ (150 μM) for 3 h and compared with untreated controls. Data from 120 cells (150 μM H_2_O_2_) and 106 cells (control) from 3 independent experiments are shown. Statistically significant differences determined by unpaired two-tailed Student's t-tests are indicated. ∗∗∗p < 0.001. Scale bar, 20 μm.

We used a combined colorimetric assay based on the conversion of (3-(4, 5-dimethylthiazolyl-2)-2, 5-diphenyltetrazolium bromide) (MTT) and the release of lactate dehydrogenase (LDH). This allows to determine both the mitochondrial activity and the cytotoxicity profile of exogenously added hydrogen peroxide for differentiated neuronal cells. We observed that a short (3 h) treatment with 150 μM hydrogen peroxide affected neither MTT conversion, as a measure of metabolic activity, nor LDH release, as a measure of cytotoxicity (Fig. 1A). In contrast, an increase to 225 μM hydrogen peroxide induced both a decrease in MTT conversion and an increase in LDH release, both of which became highly significant at 450 μM. To quantify changes in the amount of intracellular ROS, we used a commercial fluorometric assay with a cell-permeable dye that reacts with ROS and generates a fluorescence signal (abcam, ab186028). Treatment with subtoxic 150 μM hydrogen peroxide increased intracellular ROS level by ∼30 %, while toxic 450 μM hydrogen peroxide resulted in an increase of ∼70 % (Fig. 1B).

In further experiments, we therefore decided to focus on treating neuronal cells with the subtoxic concentration of 150 μM hydrogen peroxide, which results in only a moderate increase in intracellular ROS levels, likely reflecting “oxidative eustress”. Because neuronal development and function critically depend on the regulation of microtubule polymerization, we determined the effect of hydrogen peroxide on axonal microtubule dynamics using a previously established live cell imaging approach. The method is based on fluorescence decay after photoactivation (FDAP) measurements of model neurons transfected to express PAGFP-tagged α-tubulin (PAGFP-α-tubulin). Changes in microtubule dynamics were then monitored by FDAP measurements on cells in which the fluorescence of PAGFP was focally activated in the middle of an axon-like process (Fig. 1C). The FDAP curves showed that hydrogen peroxide treatment resulted in an increased fluorescence decay, indicating a higher amount of diffusing tubulin dimers and thus a lower proportion of polymerized tubulin (Fig. 1D). The application of a previously developed refined reaction-diffusion model to the FDAP data enabled the determination of the rate constants for binding (k∗_on_) and dissociation (k_off_) of PAGFP-α-tubulin at the microtubule ends in the cellular process [20]. Notably, we observed a greatly reduced k∗_on_ value, indicating that hydrogen peroxide reduces microtubule growth, and a significant increase in k_off_, indicating higher microtubule dynamics as it reflects a reduced residence time of tubulin dimers in the polymer (Fig. 1E). As a result, the amount of polymerized tubulin in the axon-like processes decreased by ∼10 % from over 80 % to ∼70 % (Fig. 1F).

Taken together, the data indicate that subtoxic concentrations of hydrogen peroxide modulate the properties of the axonal microtubule array by reducing the proportion of microtubule polymer and increasing their dynamics.

### 3.2 The mitochondria-targeted antioxidant SkQ1 and the microtubule stabilizer epothilone D prevent hydrogen peroxide-induced microtubule depolymerization

A major source of ROS under physiological conditions is mitochondria. Through their NAD^+^ pool, they play a predominant role in defining cellular responses to stress. While the mitochondrial NAD^+^/NADH redox ratio is maintained separately, it appears to be strongly connected to the cytosolic NAD^+^ pool and responsive to changes in its perturbations [38]. Therefore, we asked whether targeting the redox status of mitochondria would influence hydrogen peroxide-mediated modulation of microtubule dynamics.

To modulate the mitochondrial redox state, we used the mitochondria-targeted antioxidant SkQ1. SkQ1 is a derivative of plastoquinone, a potent antioxidant [39] and has already been used in preclinical studies to treat cardiovascular and renal diseases [40]. Indeed, treatment with SkQ1 completely abolished the hydrogen-peroxide induced increase in intracellular ROS levels (Fig. 1G). The same was true for the hydrogen peroxide-induced modulation in microtubule polymer, where pretreatment with SkQ1 completely abolished the effect of hydrogen peroxide on microtubule polymer reduction in axon-like processes (Fig. 1H and I). In contrast, TPP lacking the antioxidant quinone moiety had no effect (Fig. 1I) confirming the antioxidant activity of SkQ1 in preserving the state of axonal microtubules.

If the cytosolic redox state indeed modulates axonal microtubule dynamics, thereby leading to reduced microtubule polymer, a microtubule stabilizer may prevent this effect. To test this hypothesis, we used the well-characterized microtubule-targeting agent (MTA) epothilone D (EpoD). This small molecule microtubule stabilizer binds to the β-tubulin subunit on the luminal surface of microtubules, induces tubulin polymerization similar to paclitaxel [41], and reduces microtubule-dependent spine loss in an Alzheimer's disease model already at subnanomolar concentrations [42]. In fact, treatment with nanomolar EpoD abolished the decrease in microtubule polymer caused by hydrogen peroxide (Fig. 1J and K).

Modulation of the microtubule polymer and microtubule dynamics could affect neuronal process formation. To determine whether subtoxic hydrogen peroxide alters the morphology of the model neurons, we determined its effect on process length, process number, and cell body radius. Hydrogen peroxide had no effect on the mean process number per cell, but caused a reduction in total and mean process length by about 35 %. It also caused a reduction in cell body radius by about 20 % (Fig. 1L).

Thus, the data indicate that the mitochondrial redox state modulates microtubule dynamics in the axonal compartment and that subtoxic concentrations of hydrogen peroxide affect neuronal morphology by shortening the length of cell processes. Furthermore, the data show that microtubule-stabilizing drugs override the modulation of microtubule dynamics by the cytosolic redox state.

### 3.3 Subtoxic hydrogen peroxide modulates the structure of the axonal microtubule array and reduces microtubule mass

Axonal microtubules are not continuous but are organized as an array of relatively short microtubules, the length distribution of which appears to vary depending on the neuron type and species (reviewed in Ref. [43]). In developing mammalian axons, the average microtubule length is only a few micrometers [44]. The question therefore arises whether hydrogen peroxide not only affects microtubule dynamics and polymer amount, but also changes the microtubule arrangement in axons.

**Fig. 2:**
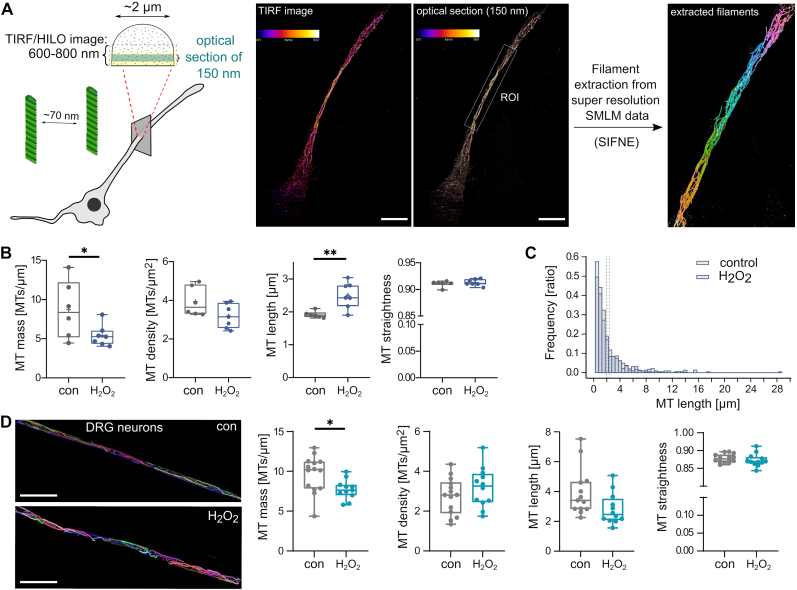
Subtoxic hydrogen peroxide modulates the structure of the axonal microtubule array and reduces microtubule mass. **A.** Shown on the left is a schematic representation of the single molecule localization microscopy (SMLM) approach to quantify MT organization. An indication of the average microtubule spacing in axon-like processes of differentiated PC12 is included. A total internal reflection fluorescence (TIRF) image in the highly inclined and laminated optical sheet (HILO) mode of the 3D-rendered SML data and a 150 nm optical section with fire color code are shown in the middle. Microtubule filaments as extracted from a selected ROI using SIFNE (single-molecule localization microscopy image filament network extractor) are shown in the rainbow color code on the right. Scale bar 10 μm. **B.** Boxplots showing the mass, density, mean length and straightness of microtubules. Each data point represents the average of a single cell (control, 6 cells with n = 902 individual microtubules; H_2_O_2_, 7 cells with n = 457 individual microtubules). H_2_O_2_ treatment (150 μM for 3 h) reduces microtubule mass and increases mean microtubule length. Statistically significant differences determined by unpaired two-tailed Student's t-tests are indicated. ∗p < 0.05, ∗∗p < 0.01. **C.** Distribution of microtubule lengths in a relative abundance histogram. Dotted lines show mean microtubule length under control conditions and with H_2_O_2_. The bin width was set to 0.5 μm and the area of the histogram bars is 1.0. H_2_O_2_ shifts the length distribution by reducing the proportion of short microtubules (0.5–2.5 μm length) and increasing the number of long microtubules (>8 μm). **D.** Extracted microtubules from an axon section of a SMLM image of H_2_O_2_-treated dorsal root ganglion (DRG) neurons compared to a control cell are shown on the left. Boxplots on the right show that treatment with 150 μM H_2_O_2_ for 3 h reduces microtubule mass but does not alter microtubule density, mean length, and straightness compared to an untreated control group. Each data point represents the average of a single cell (control, 13 cells with n = 378 individual microtubules; H_2_O_2_, 12 cells with n = 427 individual microtubules). Statistically significant differences determined by unpaired two-tailed Student's t-tests are indicated. ∗p < 0.05. Scale bar, 5 μm.

To determine the organization of the microtubule array in axon-like processes, we used single molecule localization microscopy (SMLM) using DNA-PAINT (Point Accumulation in Nanoscale Topography) [45], followed by algorithm-based filament extraction [28] (Fig. 2A). We have previously validated the approach in axon-like processes of PC12 cells and dendrites of primary neurons [17]. As expected from the FDAP data, hydrogen peroxide resulted in reduced microtubule polymer, as evidenced by lower microtubule mass (Fig. 2B, left). Interestingly, hydrogen peroxide increased the mean microtubule length in processes of PC12 cells (Fig. 2B, middle), suggesting a trend toward fewer but longer microtubules in the processes. The stiffness of the microtubules, indicated by their straightness, did not change (Fig. 2B, right). To determine how hydrogen peroxide affects microtubule length, we plotted the length distribution in a relative frequency histogram (Fig. 2C). The data show that hydrogen peroxide increases mean microtubule length primarily by reducing the proportion of short microtubules (0.5–2.5 μm length).

PC12 cells are widely used as a model for peripheral neurons and to study mechanisms of neuronal differentiation. To determine whether hydrogen peroxide has a similar effect on microtubules in primary neurons of the peripheral nervous system (PNS), we used dissociated dorsal root ganglion (DRG) cultures from adult mice. The cultures were treated with 150 μM hydrogen peroxide similarly to PC12 cells and the organization of the microtubule array in the axons was determined by SMLM using DNA-PAINT. Microtubule mass in DRG axons was very similar to that of PC12 cells and hydrogen peroxide also resulted in a significant reduction in their mass (Fig. 2D, left panel). However, mean microtubule length was much higher in DRG neurons and hydrogen peroxide did not alter the mean length of the microtubule population (Fig. 2D, middle). Microtubule stiffness, as measured by their straightness, was generally lower in DRG neurons than in PC12 cells, but also did not change after treatment with hydrogen peroxide (Fig. 2D, right panel).

Thus, the data suggest that hydrogen peroxide alters the organization of the microtubule array in axon-like processes of model neurons and in authentic axons of primary neurons, thereby reducing the number of microtubules per micrometer. However, the effect on microtubule length appears to differ depending on the cell type and might depend on the microtubule length distribution.

### 3.4 Hydrogen peroxide-induced changes in the microtubule array reduce the proportion of mobile vesicles but have no effect on the speed and velocity of vesicle transport

A change in axonal microtubule arrangement may have a direct impact on the properties of axonal transport, as *C. elegans* motor neurons have been shown to pause axonal transport at the ends of microtubules before switching to a new polymer [46]. This suggests that altering microtubule tracks can affect the efficiency of axonal transport.

**Fig. 3:**
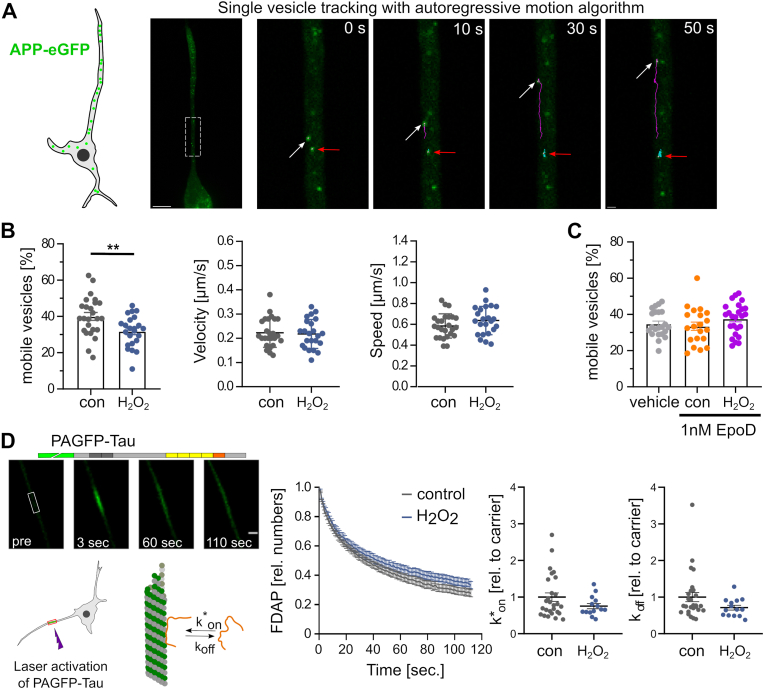
Hydrogen peroxide-induced changes in the microtubule array reduce the proportion of mobile vesicles but have no effect on the speed and velocity of vesicle transport, or the interaction of the axonal tau protein with microtubules. **A.** Tracking APP vesicles in a PC12 cell process using eGFP-tagged APP and an autoregressive motion algorithm. The first micrograph shows an overview of a cell expressing APP-eGFP. The images on the right show selected times of the APP vesicle movement from the part of the process marked by the white box in the overview image. Arrows point to a moving (white) and a stationary vesicle (red). Scale bars, 10 μm (overview) and 1 μm (time lapse). **B.** The bar graph shows proportions of mobile vesicles in the axon-like process under control conditions and in response to H_2_O_2_. Velocity and speed of mobile vesicles are shown in the scatter plots on the right (mean ± SEM of n = 25 cells with 1595 trajectories (control) and 23 cells with 1209 trajectories (H_2_O_2_); each point represents an average value for one analyzed cell). Statistically significant differences determined by unpaired two-tailed Student's t-tests are indicated. ∗∗p < 0.01. **C.** Fraction of mobile vesicles in cells pretreated with 1 nM EpoD according to the timeline shown in Fig. 2D. Comparison with vehicle (0.01 % DMSO) shows no effect of EpoD on the fraction of mobile vesicles. Each data point represents an average value for a corresponding cell (mean ± SEM of 20–25 cells with 1276–1720 trajectories is shown). **D.** Effect of H_2_O_2_ on the interaction of tau with microtubules. A schematic representation of the FDAP approach and the expressed tau construct is shown on the left. FDAP plots after photoactivation of PAGFP-Tau expressing cells (middle) show a similar fluorescence decay with and without H_2_O_2_. Likewise, the scatterplots of the association (k∗_on_) and dissociation rate constants (k_off_) (right) show no statistically significant differences. Mean values ± SEM of 25 (control) and 14 (H_2_O_2_) cells are shown.

To determine whether the hydrogen peroxide-induced change in the microtubule array affects axonal transport parameters, we used single-vesicle tracking of eGFP-tagged amyloid precursor protein (APP), a key axonal transport cargo [47] (Fig. 3A). Hydrogen peroxide treatment reduced the proportion of mobile vesicles by approximately 20 % compared to a control (Fig. 3B, left), indicating a reduction in the total amount of cargo transported. To quantify the movement of mobile vesicles, we determined the effect of hydrogen peroxide on velocity (displacement per time) and speed (run length per time) from the trajectories of each cell analyzed (Fig. 3B, middle and right). Both the velocity and speed of APP-vesicles were the same under both conditions, indicating that the change in the number of microtubules had no effect on the transport of the vesicles once they were moving.

To confirm that the observed reduction in the proportion of mobile vesicles is due to the change in the organization of the microtubule array, we determined the effect of the microtubule stabilizer EpoD. We previously observed that nanomolar concentrations of EpoD shifted the length distribution of microtubules in axon-like processes toward shorter and denser microtubules [17]. Indeed, EpoD abolished the effect of hydrogen peroxide on reducing the fraction of mobile vesicles (Fig. 3C), suggesting that the effect was caused by the altered organization of the microtubule array.

Thus, the data indicate that hydrogen peroxide has the potential to influence the efficiency of axonal transport by reducing the total amount of cargo transported, likely due to its effect on modulating the organization of the microtubule array.

### 3.5 Hydrogen peroxide-induced changes in the microtubule array do not affect the dynamic interaction of the axonal tau protein with microtubules

Tau protein is an axonally enriched microtubule-associated protein (MAP) that is thought to regulate axonal microtubule polymerization. Pathological changes in tau are involved in a class of neurodegenerative diseases collectively referred to as tauopathies [5]. Under physiological conditions, tau dynamically interacts with microtubules and exhibits a “kiss and hop” behavior that is likely required to regulate microtubule polymerization without affecting the efficiency of axonal transport [48]. Indeed, post-translational modifications of tau that make tau microtubule interaction less dynamic can lead to axonal transport defects that cause dendritic atrophy in tauopathies [21,49].

Therefore, hydrogen peroxide could induce changes in the interaction of tau with axonal microtubules, which may affect microtubule dynamics, cause the observed reduction in microtubule polymer, and impair microtubule-dependent cargo transport. We used FDAP experiments to determine a possible change in the dynamics of tau interaction with microtubules in axon-like processes of the model neurons. Tau was N-terminally tagged with photoactivatable GFP (PAGFP) and expressed exogenously in PC12 cells, which were differentiated to a neuronal phenotype. Following focal activation of tau in hydrogen peroxide-treated or control cells, tau showed similar dissipation from the activation area (Fig. 3D, left). Application of a refined reaction-diffusion model of the tau-microtubule interaction allowed the determination of the binding constants (k∗_on_ and k_off_ rates) of the tau microtubule-interaction [20]. We did not detect any difference in either kinetic constant as a result of hydrogen peroxide treatment (Fig. 3D, right).

Thus, the data indicate that hydrogen peroxide does not affect the dynamics of tau-microtubule interaction in axon-like processes. Consequently, the data also indicates that the increase in microtubule dynamics as a result of hydrogen peroxide treatment is not caused by a change in the interaction of tau with microtubules.

### 3.6 Subtoxic hydrogen peroxide modulates the phosphorylation state of microtubule-regulating proteins

**Fig. 4:**
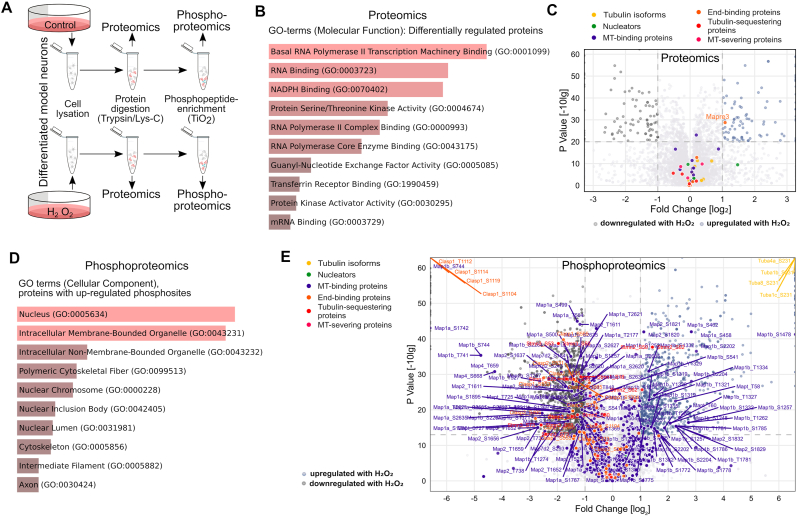
Subtoxic hydrogen peroxide modulates the phosphorylation state of microtubule-regulating proteins. **A.** Schematic representation of the approach for proteomics and phosphoproteomics analyses of differentiated model neurons treated with hydrogen peroxide compared to control. **B.** Bar plot with the top enriched GO-terms for molecular function of all differentially regulated proteins in response to H_2_O_2_. The length of the bar indicates the p-value, reflecting the enrichment significance of each term. Note an enrichment of proteins mostly involved in RNA processing and redox modulation. **C.** Volcano plot showing up- and down-regulated proteins in hydrogen peroxide-treated cultures compared to controls. Log_2_ fold changes are plotted against -log10 p-values. Significant upregulation upon H_2_O_2_ treatment is shown in blue, downregulation in dark grey. Members of different groups of microtubule-related proteins are indicated. The axes are cut for representation purposes (x-axis from −3 to 3, y-axis from 0 to 60); all the proteins above the limits are shown as points at each limit border. Members of different groups of microtubule-related proteins are indicated in different colors, with significantly changed ones labelled with their gene names. Only one protein of the more than 30 identified tubulin and microtubule-regulating proteins (MAPRE3) shows a slight upregulation. **D.** Bar plot with the top enriched GO-terms for cellular components of all proteins with upregulated phosphosites in response to H_2_O_2_. The length of the bar indicates the p-value, reflecting the enrichment significance of each term. Note enrichment of nuclear components, organelles and proteins of the cytoskeleton. **E.** Volcano plot showing up- and down-regulated phosphosites in hydrogen peroxide-treated cultures compared to controls. The axes are cut for representation purposes and all phosphosites above the limits are shown as points at each limit border. Coloring and labelling as in C.

Hydrogen peroxide, as a second messenger molecule, is thought to link redox biology to intrinsic signalling pathways [12]. Thus, it is likely that subtoxic hydrogen peroxide shapes the axonal microtubule array by affecting the expression or phosphorylation of tubulin or microtubule-regulating proteins. To identify the respective target molecules and their modification, we performed proteomics and phosphoproteomics analysis of differentiated model neurons treated with hydrogen peroxide compared to control conditions (Fig. 4A). GO-term analysis for differentially expressed proteins revealed mainly NADPH-binding and RNA polymerase-binding proteins (Fig. 4B). The only microtubule-regulating protein with differential expression was MAPRE3 (Microtubule-associated protein RP/EB family member 3, EB3), which showed slightly increased expression in hydrogen peroxide-exposed cells (Fig. 4C). To validate the change in EB3 levels, we performed Western blot analysis under identical conditions. The data showed no significant change in EB3 levels (0.757 ± 0.145 (SEM) with hydrogen peroxide versus 1.0 ± 0.053 (SEM) for the control; n = 8).

In contrast to the moderate effect on the expression of microtubule-regulating proteins, hydrogen peroxide had a strong effect on their phosphorylation, as determined by phosphoproteomic analysis (Fig. 4D and E). GO-term analysis of proteins with upregulated phosphosites revealed the presence of many cytoskeletal proteins, particularly components of the microtubule skeleton (Fig. 4D). Further examination of the proteins with upregulated phosphosites revealed that several functional subgroups of microtubule-regulating proteins, particularly microtubule-associated and tubulin-sequestering proteins, exhibited increased phosphorylation (Fig. 4E).

Thus, the data indicate that hydrogen peroxide shapes the axonal microtubule array primarily by modulating the phosphorylation status of different functional groups of microtubule-regulating proteins.

### 3.7 Hydrogen peroxide and arsenite induce a specific phosphorylation signature of MAP1B as a major target of microtubule-regulating proteins

If hydrogen peroxide acts as a specific second messenger molecule, it would be expected to selectively regulate signaling pathways that are distinct from the action of other stressors. Therefore, we decided to compare the effect of subtoxic hydrogen peroxide with the effect of arsenite, a naturally occurring toxicant commonly used as a cellular stress inducer [[50], [51], [52]].

**Fig. 5:**
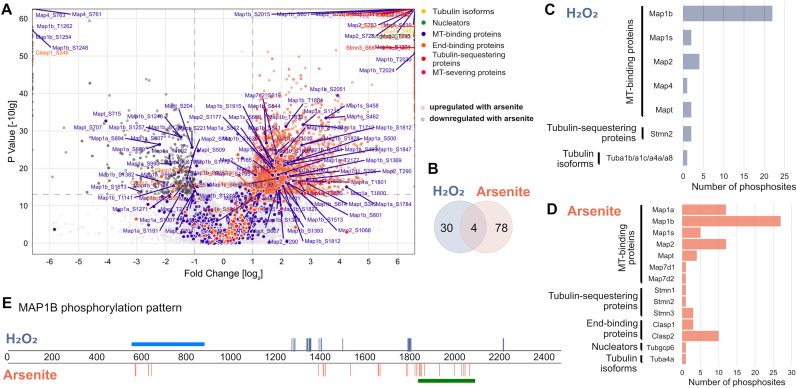
Hydrogen peroxide and arsenite induce a specific phosphorylation signature of MAP1B as a major target of microtubule-regulating proteins. **A.** Volcano plot showing upregulated phosphosites in arsenite-treated cells compared to control. Members of different groups of microtubule-regulating proteins are indicated by the same color code as in Fig. 4C. Log_2_ fold changes are plotted against -log10 p-values. Significant upregulation upon arsenite treatment is shown in orange, downregulation in dark grey. The axes are cut for representation purposes and all phosphosites above the limits are shown as points at each limit border. **B.** Venn diagram showing low overlap of phosphosites of microtubule-related proteins upregulated in response to hydrogen peroxide or arsenite. **C, D.** Distribution of upregulated phosphosites on different microtubule-related proteins in response to hydrogen peroxide (C) or arsenite (D). Note that MT-binding proteins and especially MAP1B are the main target. A list of all altered phosphorylation sites in microtubule-regulating proteins can be found in Supplementary Table 1 (hydrogen peroxide) and Supplementary Table 2 (arsenite). **E.** Graphical representation of the different phosphosites on MAP1B that are upregulated in response to hydrogen peroxide or arsenite. The blue bar shows the microtubule-binding region according to the deletion study by Ref. [80]. Phosphorylated epitopes recognized by the monoclonal antibody SMI-31 [71], which detects disease-associated mode I phosphorylation sites, which cause a loss of the microtubule stabilizing activity, are indicated by the dark green bar.

Phosphoproteomic analysis showed that arsenite also affected the phosphorylation of several functional groups of microtubule-regulating proteins, including microtubule-binding proteins, microtubule nucleators, and tubulin-binding proteins (Fig. 5A). In fact, arsenite induced a much higher number of upregulated phosphosites than hydrogen peroxide (Fig. 5B). Notably, the overlap of the upregulated phosphosites in microtubule-related proteins (tubulin isoforms and microtubule-regulating proteins) induced by hydrogen peroxide and arsenite was small, and less than 5 % (4 out of 82) of the phosphosites increased by arsenite were also increased in the presence of hydrogen peroxide.

A comparison of the target proteins that showed increased phosphorylation revealed that twice as many microtubule-related proteins were altered in arsenite-exposed cells (Fig. 5C and D; a list of all altered phosphorylation sites in microtubule-regulating proteins can be found in Supplementary Table 1 (hydrogen peroxide) and Supplementary Table 2 (arsenite)). In both cases, the microtubule-binding protein MAP1B was the main target. MAP1B is predominantly expressed in the nervous system and has been implicated in the regulation of axonal elongation and guidance [53,54]. MAP1B showed a complex pattern of increased phosphorylation sites with both hydrogen peroxide (22 sites) and arsenite (27 sites) (Fig. 5E). Notably, only one of the sites (S1772) showed increased phosphorylation under both conditions. This specificity of increased phosphorylation at selected sites was also present in other MAPs such as tau (total of 6 phosphosites, no overlap) and MAP2 (total of 16 phosphosites, no overlap).

Thus, the data indicate that hydrogen peroxide leads to a specific phosphorylation pattern of microtubule-regulating proteins that is largely distinct from the effects of arsenite as an alternative stressor though arsenite increases the phosphorylation of a much larger number of phosphorylation sites. The data also show that MAP1B phosphorylation is a major target for both stressors, creating a stress-specific phosphorylation signature on this protein.

### 3.8 Cell-wide phosphoproteome analysis of predicted upstream kinases in neuronally differentiated cells reveals a pattern of inversely regulated kinases by hydrogen peroxide and arsenite

**Fig. 6:**
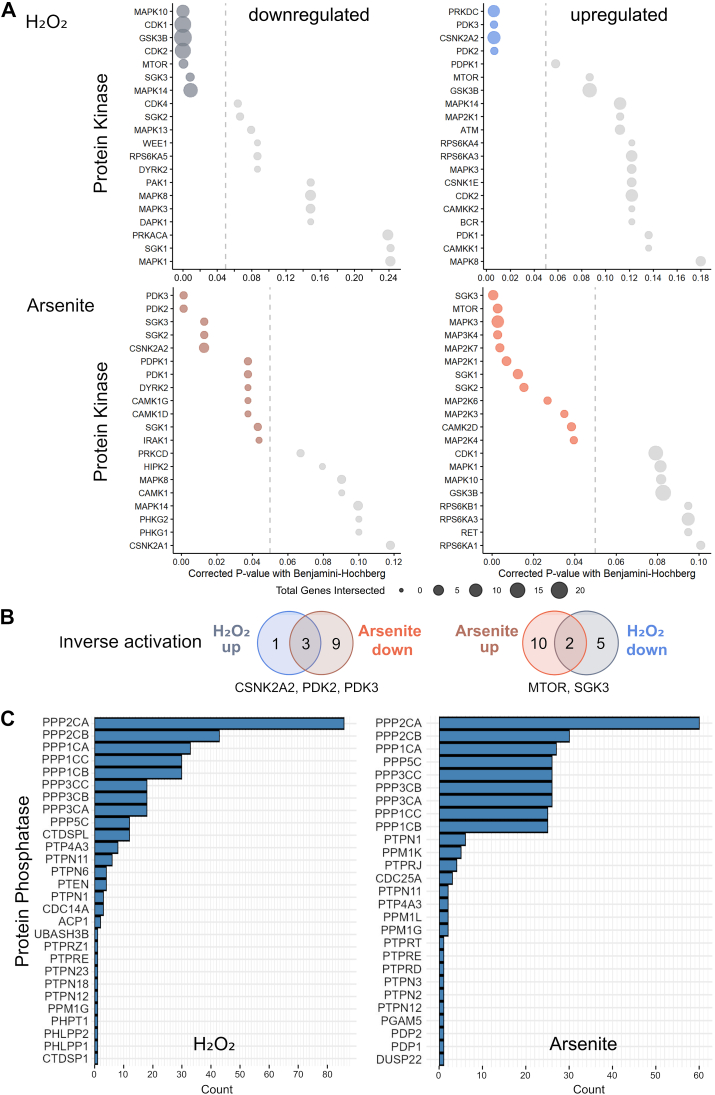
Cell-wide phosphoproteome analysis of predicted upstream kinases in neuronally differentiated cells reveals a pattern of inversely regulated kinases by hydrogen peroxide and arsenite. **A.** Kinase enrichment analysis of the phosphoproteomics data to identify the pattern of kinases responsible for increased phosphorylation of all cellular proteins in response to hydrogen peroxide (left) and arsenite (right). Note that there is no overlap between significantly upregulated upstream kinases with hydrogen peroxide and arsenite. **B.** Venn diagrams showing upstream kinases leading to a reverse change in phosphorylation, i.e. induction of increased phosphorylation with hydrogen peroxide and reduced phosphorylation with arsenite (top) and increased phosphorylation with arsenite and reduced phosphorylation with hydrogen peroxide (bottom). The respective upstream kinases with reverse change are indicated below the corresponding Venn diagram. **C.** Phosphatase mapping to identify specific phosphatase activity based on the dephosphorylated residues of all proteins in response to hydrogen peroxide and arsenite. Note that the three highest ranked protein phosphatases identified are the same after treatment with hydrogen peroxide and arsenite.

The fact that hydrogen peroxide and arsenite have a different effect on the phosphorylation of microtubule-regulating proteins suggests that they influence specific signaling cascades differently. Therefore, we performed a kinase enrichment analysis using all altered phosphosites as input to predict the upstream kinases responsible for the different phosphorylation events observed [55]. Notably, we observed no overlap between the predicted upstream kinases activated by hydrogen peroxide and arsenite (Fig. 6A). The upstream kinases activated by hydrogen peroxide included the DNA-dependent protein kinase PRKDC, the pyruvate dehydrogenase kinases PDK2 and 3, and the casein kinase 2 subunit CSNK2A2. On the other hand, prominent predicted upstream kinases activated by arsenite were several members of the MAP kinase signal transduction pathway (MAPK3, MAP3K4, MAP2K1, 3, 4, 6, and 7), mammalian target of rapamycin (mTOR), and serum/glucocorticoid regulated protein kinases SGK1, 2 and 3. Several kinases even showed inverse activation with hydrogen peroxide and arsenite. These included pyruvate dehydrogenase kinases (PDKs), mTOR, and glucocorticoid-regulated kinase 3 (SGK3) (Fig. 6B).

Changes in the phosphoproteome could also be related to phosphatase inhibition induced by the stressors. To identify a possible contribution of phosphatases, we performed phosphatase mapping to identify the specific phosphatase activity based on the dephosphorylated residues of all proteins in response to hydrogen peroxide and arsenite. We observed that the three highest ranked protein phosphatases after hydrogen peroxide and arsenite treatment were the same and included the serine/threonine-protein phosphatase 2A catalytic subunit alpha and beta (PPP2CA, PPP2CB) and the serine/threonine-protein phosphatase PP1-alpha (PPP1CA). In fact, 9 out of 10 enzymes in the top ten list were present with hydrogen peroxide and arsenite (Fig. 6C).

Taken together, cell-wide phosphoproteome analysis shows that different signaling pathways are activated by hydrogen peroxide and arsenite, with hydrogen peroxide predominantly activating the DNA-dependent protein kinase PRKDC, the pyruvate dehydrogenase kinases PDK2 and 3, and the casein kinase 2 subunit CSNK2A2 as upstream kinases. This may indicate that these pathways in particular play a central role in physiological redox signaling, including the modulation of axonal microtubule organization.

## 4 Discussion

Reactive oxygen species are involved in a variety of physiological cellular functions such as cell proliferation, differentiation and maturation [56]. However, loss of ROS homeostasis can lead to oxidative distress and promote pathological conditions in the brain [6]. The development, maturation and maintenance of neurons critically depends on the microtubule cytoskeleton. Changes of microtubules, microtubule-regulating proteins and microtubule-dependent functions such as axonal transport occur early in the disease process and disruption of microtubule dynamics may be a key mechanism contributing to neurodegeneration [1] and neuronal aging [57]. However, it is largely unknown how physiological, nontoxic ROS levels (oxidative eustress) influence axonal microtubule dynamics and microtubule-dependent functions.

Here, we show that (1) a subtoxic concentration of the redox messenger hydrogen peroxide increases microtubule dynamics and shapes microtubule organization in axon-like processes, that (2) hydrogen peroxide modulates the phosphorylation state of different functional groups of microtubule-regulating proteins, and that (3) subtoxic hydrogen peroxide induces a complex phosphorylation pattern of MAP1B as a major target protein, which differs from arsenite as an alternative stress inducer. By cell-wide phosphoproteome analysis, we further provide evidence that (4) different signaling pathways are inversely activated by hydrogen peroxide and arsenite. In particular, hydrogen peroxide treatment was associated with signaling pathways that suppress apoptosis and regulate brain metabolism (PRKDC, CK2, PDKs), while treatment with arsenite led to the activation of pathways related to neurodegeneration (mTOR, SGKs).

**Fig. 7:**
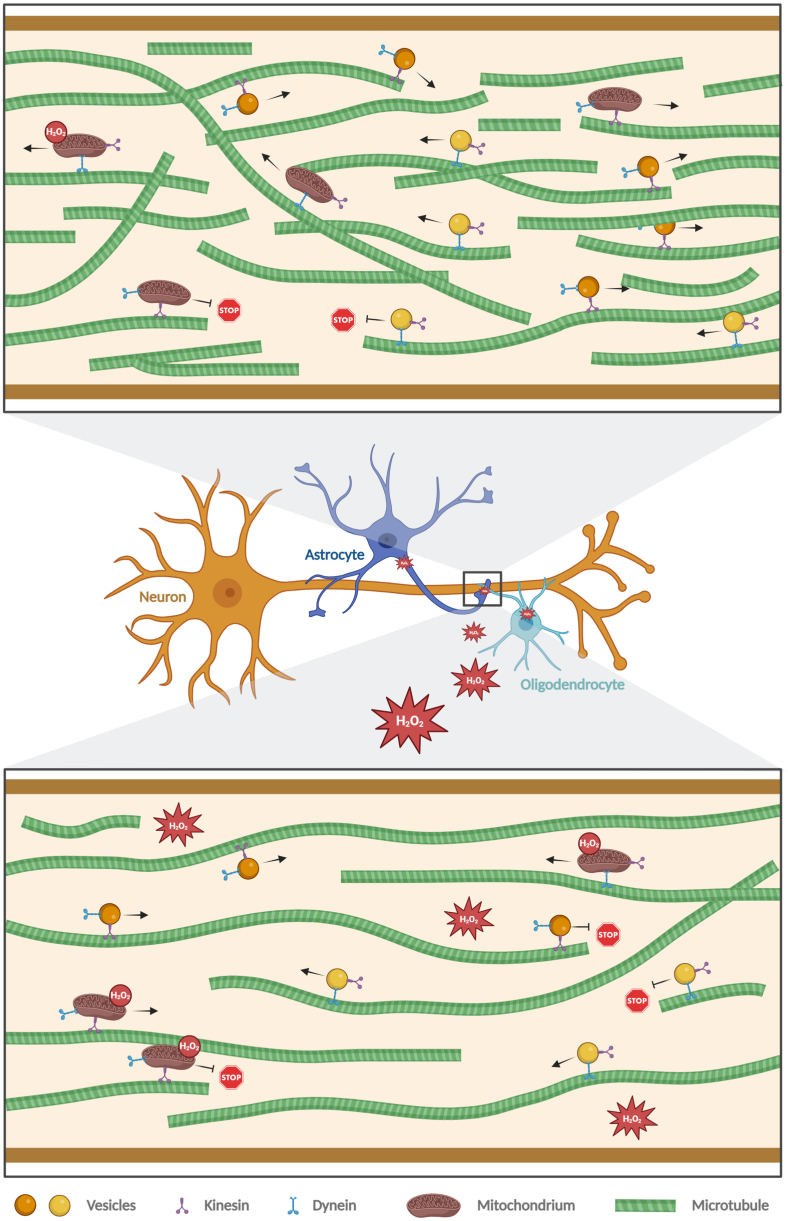
Schematic representation of the effect of hydrogen peroxide on axonal microtubule organization and microtubule-dependent transport. Hydrogen peroxide diffuses or distributes through cells and tissues by passive transport and can reach neighboring axons when produced in oligodendrocytes or astrocytes. In addition, it is also produced by mitochondria in the axons. In axons, H_2_O_2_ causes a reorganization of the microtubule cytoskeleton toward a lower microtubule mass, which reduces the efficiency of vesicle transport. Figure created with BioRender.com.

Axonal microtubules exhibit a unique organization in that they exist in relatively short fragments with a uniform orientation and their dynamic plus-ends pointing towards the axon tip. Although most axonal microtubules are more stable than the microtubules of dendrites, axons also possess dynamic microtubules that undergo phases of polymerization and depolymerization, a process known as dynamic instability [58]. The dynamic microtubules in the axon can act as sensors of the cellular microenvironment and enable the rapid reorganization of the cytoskeleton in response to changes in the environment [59]. The regulation of microtubule nucleation and the dynamics of their polymerization could therefore play an important role in local axon homeostasis for axon maintenance, function and pathology [2]. Regulation by ROS can thus couple neuronal activity to the local organization of the microtubule array. High neuronal activity would lead to an increase in ROS species due to more active mitochondria. A physiologically increased hydrogen peroxide content would then lead to a rearrangement of the microtubule array by decreasing microtubule density, accompanied by a reduced amount of transported cargo, as we observed in our experiments (Fig. 7). This would create a negative feedback loop to dampen over-activation of the neuron.

Hydrogen peroxide may also play a role in communication between glial cells and neurons. As a messenger molecule, hydrogen peroxide diffuses or distributes through cells and tissues by passive transport, which would allow myelinating oligodendrocytes, which are known to provide metabolic and functional support to the underlying axon [60,61], to modulate the structure and dynamics of axonal microtubules. Oligodendrocytes, in turn, could also act as a sink for hydrogen peroxide produced locally in the axon. Therefore, it would be interesting to determine the possible crosstalk of oligodendrocytes and neurons with respect to redox signaling.

Our data show that several functional groups of microtubule-regulating proteins are differentially phosphorylated due to hydrogen peroxide signaling. By far the largest group is microtubule-associated proteins (MAPs), followed by the tubulin sequestering protein stathmin 2. This suggests that the other groups of microtubule-regulating proteins such as microtubule-severing factors, end-binding proteins or microtubule nucleators are not involved in the immediate response to hydrogen peroxide with regard to axonal microtubule homeostasis. Interestingly, microtubule-binding proteins and tubulin-sequestering proteins were also identified as the most important drivers for the development of increased neuronal complexity during vertebrate evolution, as they showed the largest increase in the number of orthologs and predicted protein-coding splice variants [32]. Thus, MAPs and tubulin-sequestering factors appear to be the most important microtubule-regulating proteins in adapting the microtubule skeleton to changes in the environment, both on an evolutionary scale and in terms of regulating axonal microtubule homeostasis. With arsenite treatment, end-binding proteins and a microtubule nucleator also joined the functional groups modified by phosphorylation, which may indicate that increased phosphorylation of end-binding proteins in particular might contribute to the changes of the axonal microtubule array under toxic conditions.

Our data show that MAP1B is a major target of phosphorylation during both hydrogen peroxide and arsenite treatment. MAP1B appears to preferentially associate with tyrosinated (dynamic) microtubules, rather than detyrosinated (stable) microtubules [62], which may increase the pool of dynamic microtubules [63,64]. Such behavior could be important for the local regulation of axonal microtubule dynamics and polymerization. Phosphorylation of MAP1B at different sites can regulate the local fine-tuning of neurite branching and microtubule dynamics [[65], [66], [67]]. Historically, two types of MAP1B phosphorylation have been distinguished. Mode I phosphorylation induces a shift in electrophoretic mobility and decreases with development, while mode II phosphorylation does not affect electrophoretic mobility and remains unchanged [68]. Phosphorylation at mode I sites results in a loss of microtubule-stabilizing ability [69] and mode I phosphorylated MAP1B is also observed in neurofibrillary tangles (NFTs) and dystrophic neurites in Alzheimer's disease brains [70]. This suggests that mode I phosphorylation may be associated with nervous system pathogenesis. In contrast, mode II phosphorylation is mediated for example by casein kinase 2 [67], a predicted upstream kinase with inverse regulation with hydrogen peroxide and arsenite. Regions of phosphorylated epitopes recognized by the monoclonal antibody SMI-31 implicated in detecting mode I phosphorylation sites [71] are present both after arsenite treatment (1836–2076) and hydrogen peroxide treatment (1244–1264) (Fig. 5E). It will be interesting to determine the functional consequences of the hydrogen peroxide-induced change in phosphorylation, in order to determine sites that may be crucial for the physiological regulation of microtubule polymerization. However, the remarkable complexity of MAP1B phosphorylation makes this a difficult undertaking.

Cell-wide phosphoproteome analyses showed that different signaling pathways are activated by hydrogen peroxide and arsenite. The upstream kinases activated by hydrogen peroxide included kinases known to suppress apoptosis, such as the DNA-dependent protein kinase PRKDC [72] or the casein kinase 2 subunit CSNK2A2 [73], as well as the pyruvate dehydrogenase kinases PDK2 and 3, which are known to be involved in the regulation of brain metabolism [74]. Of particular importance for specific regulation of phosphorylation might be those signaling pathways that lead to a reverse change in phosphorylation, i.e. activation under one condition but inhibition under the other. Upstream kinases activated for hydrogen peroxide versus arsenite included pyruvate dehydrogenase kinases (PDKs), which play a crucial role in aerobic metabolism and link glycolysis to the tricarboxylic acid cycle and ATP generation [75], and a subunit of casein kinase 1, which contributes significantly to the generation of the human phospho-proteome [76]. Upstream kinases activated for arsenite versus hydrogen peroxide included mTOR and glucocorticoid-regulated kinase 3 (SGK3), kinases whose dysregulation has been linked to metabolic dysfunction and disease [77,78]. Phosphatase mapping revealed no significant differences between cells treated with hydrogen peroxide and arsenite. This suggests that the observed differences are predominantly due to changes in the activity of upstream kinases.

Modulation of phosphorylation of the microtubule-associated protein tau could be a candidate to regulate axonal microtubule polymerization and vesicle transport. Increased phosphorylation of tau at selected sites reduces its interaction with microtubules and affects the dynamics of its kiss-and-hop interaction with axonal microtubules [19]. Increased tau phosphorylation is also associated with the development of tauopathies such as Alzheimer's disease [5,79]. However, our data show that hydrogen peroxide does not affect the tau-microtubule interaction and that tau is not a major target of hydrogen peroxide-induced changes in phosphorylation. This suggests that increased tau phosphorylation is not involved in the regulation of microtubule polymerization, at least not under conditions of physiological redox signaling.

## CRediT authorship contribution statement

**Christian Conze:** Writing – original draft, Investigation. **Nataliya I. Trushina:** Writing – original draft, Investigation. **Nanci Monteiro-Abreu:** Writing – original draft, Investigation. **Lisha Singh:** Writing – review & editing, Investigation. **Daniel Villar Romero:** Writing – original draft, Investigation. **Eike Wienbeuker:** Writing – original draft, Investigation. **Anna-Sophie Schwarze:** Investigation. **Michael Holtmannspötter:** Writing – original draft, Investigation. **Lidia Bakota:** Writing – original draft, Supervision. **Roland Brandt:** Writing – review & editing, Writing – original draft, Supervision, Funding acquisition, Conceptualization.

## Declaration of competing interest

The authors declare no competing interests.

## Data Availability

Data will be made available on request.
