# Supplementary material for: Redox signaling modulates axonal microtubule organization and induces a specific phosphorylation signature of microtubule-regulating proteins
Source: Redox Biol. 2025 Apr 3;83:103626. doi: 10.1016/j.redox.2025.103626 (PMC12019850; doi:10.1016/j.redox.2025.103626)
Supplement: Multimedia component 1 [file mmc1.docx]

**Supplementary Table 1:** Differentially altered phosphorylation sites of microtubule-regulating proteins in response to hydrogen peroxide (based on individual phosphopeptides).

| **Category** | **Gene Phosphosite** | **Fold Change [log₂]** | **P Value [-10lg]** | **Change direction** |
| --- | --- | --- | --- | --- |
| MT-binding proteins | Map1b_S1248 | 2,735455037 | 16,43 | Increased |
| MT-binding proteins | Map1b_S1257 | 2,735455037 | 16,43 | Increased |
| MT-binding proteins | Map1b_S1257 | 1,868938419 | 14,91 | Increased |
| MT-binding proteins | Map1b_S1315 | 1,390673683 | 15,16 | Increased |
| MT-binding proteins | Map1b_S1319 | 1,390673683 | 15,16 | Increased |
| MT-binding proteins | Map1b_S1319 | 1,311074066 | 22,79 | Increased |
| MT-binding proteins | Map1b_S1332 | 2,074119477 | 16,23 | Increased |
| MT-binding proteins | Map1b_S1332 | 1,302028082 | 30,89 | Increased |
| MT-binding proteins | Map1b_S1371 | 1,027823275 | 15,24 | Increased |
| MT-binding proteins | Map1b_S1380 | 1,027823275 | 15,24 | Increased |
| MT-binding proteins | Map1b_S1382 | 1,486568807 | 14,25 | Increased |
| MT-binding proteins | Map1b_S1478 | Inf^1^ | 41,25 | Increased |
| MT-binding proteins | Map1b_S1772 | 1,503414138 | 14,76 | Increased |
| MT-binding proteins | Map1b_S1775 | 1,13296668 | 14,8 | Increased |
| MT-binding proteins | Map1b_S1778 | 1,13296668 | 14,8 | Increased |
| MT-binding proteins | Map1b_S1778 | 1,503414138 | 14,76 | Increased |
| MT-binding proteins | Map1b_S1785 | 1,13296668 | 14,8 | Increased |
| MT-binding proteins | Map1b_S1785 | 2,301161158 | 15,51 | Increased |
| MT-binding proteins | Map1b_S1786 | 2,281546964 | 15,39 | Increased |
| MT-binding proteins | Map1b_S2202 | 3,020425539 | 36,5 | Increased |
| MT-binding proteins | Map1b_S2202 | 1,609867736 | 13,84 | Increased |
| MT-binding proteins | Map1b_S2204 | 1,609867736 | 13,84 | Increased |
| MT-binding proteins | Map1b_S2204 | 2,841280042 | 28,03 | Increased |
| MT-binding proteins | Map1b_T1262 | 2,735455037 | 16,43 | Increased |
| MT-binding proteins | Map1b_T1262 | 1,868938419 | 14,91 | Increased |
| MT-binding proteins | Map1b_T1321 | 1,311074066 | 22,79 | Increased |
| MT-binding proteins | Map1b_T1327 | 2,074119477 | 16,23 | Increased |
| MT-binding proteins | Map1b_T1334 | 1,50510929 | 24,11 | Increased |
| MT-binding proteins | Map1b_T1781 | 2,301161158 | 15,51 | Increased |
| MT-binding proteins | Map1b_T1781 | 2,281546964 | 15,39 | Increased |
| MT-binding proteins | Map1b_Y1329 | 1,302028082 | 30,89 | Increased |
| MT-binding proteins | Map1b_Y1329 | 1,50510929 | 24,11 | Increased |
| MT-binding proteins | Map1s_S458 | 2,837489771 | 41,99 | Increased |
| MT-binding proteins | Map1s_S462 | 2,837489771 | 41,99 | Increased |
| MT-binding proteins | Map2_S1820 | 1,120346597 | 41,96 | Increased |
| MT-binding proteins | Map2_S1821 | 1,120346597 | 41,96 | Increased |
| MT-binding proteins | Map2_S1829 | 2,305597064 | 15,49 | Increased |
| MT-binding proteins | Map2_S1832 | 2,305597064 | 15,49 | Increased |
| MT-binding proteins | Map4_S231 | 1,265121086 | 15,5 | Increased |
| MT-binding proteins | Mapt_T58 | 2,26588586 | 22,47 | Increased |
| MT-binding proteins | Mapt_T60 | 2,412556883 | 17,82 | Increased |
| Tubulin-sequestering proteins | Stmn2_S50 | 1,432466259 | 37,67 | Increased |
| Tubulin-sequestering proteins | Stmn2_S62 | 1,058678118 | 25,66 | Increased |
| Tubulin-sequestering proteins | Stmn2_S62 | 1,432466259 | 37,67 | Increased |
| Tubulin isoforms | Tuba1b_S231 | Inf | 66,41 | Increased |
| Tubulin isoforms | Tuba1c_S231 | Inf | 66,41 | Increased |
| Tubulin isoforms | Tuba4a_S231 | Inf | 66,41 | Increased |
| Tubulin isoforms | Tuba8_S231 | Inf | 66,41 | Increased |

^1^Inf: Detected only with hydrogen peroxide

| **Category** | **Gene Phosphosite** | **Fold Change [log₂]** | **P Value [-10lg]** | **Change direction** |
| --- | --- | --- | --- | --- |
| End-binding proteins | Clasp1_S1104 | -Inf^2^ | 87,33 | Decreased |
| End-binding proteins | Clasp1_S1104 | -1,075737654 | 13,74 | Decreased |
| End-binding proteins | Clasp1_S1114 | -Inf | 86,99 | Decreased |
| End-binding proteins | Clasp1_S1119 | -Inf | 87,33 | Decreased |
| End-binding proteins | Clasp1_S548 | -1,200146076 | 13,79 | Decreased |
| End-binding proteins | Clasp1_S553 | -2,488329559 | 29,04 | Decreased |
| End-binding proteins | Clasp1_S555 | -1,200146076 | 13,79 | Decreased |
| End-binding proteins | Clasp1_S555 | -1,780593792 | 39,76 | Decreased |
| End-binding proteins | Clasp1_T1112 | -Inf | 86,99 | Decreased |
| End-binding proteins | Clasp2_S1004 | -1,333882729 | 28,17 | Decreased |
| End-binding proteins | Clasp2_S1005 | -1,333882729 | 28,17 | Decreased |
| End-binding proteins | Clasp2_S326 | -1,031409331 | 13,58 | Decreased |
| End-binding proteins | Clasp2_S331 | -1,44105147 | 19,7 | Decreased |
| End-binding proteins | Clasp2_S333 | -1,031409331 | 13,58 | Decreased |
| End-binding proteins | Clasp2_S333 | -1,44105147 | 19,7 | Decreased |
| End-binding proteins | Clasp2_S495 | -2,79404279 | 17,58 | Decreased |
| End-binding proteins | Clasp2_S531 | -1,406052425 | 28,58 | Decreased |
| End-binding proteins | Clasp2_S535 | -1,406052425 | 28,58 | Decreased |
| MT-binding proteins | Map1a_S1280 | -1,617314353 | 16,82 | Decreased |
| MT-binding proteins | Map1a_S1594 | -1,500790052 | 21,85 | Decreased |
| MT-binding proteins | Map1a_S1622 | -1,004039937 | 16,82 | Decreased |
| MT-binding proteins | Map1a_S1742 | -Inf | 43,05 | Decreased |
| MT-binding proteins | Map1a_S1763 | -1,132698139 | 14,95 | Decreased |
| MT-binding proteins | Map1a_S1767 | -1,132698139 | 14,95 | Decreased |
| MT-binding proteins | Map1a_S1895 | -1,573155294 | 20,42 | Decreased |
| MT-binding proteins | Map1a_S1897 | -1,612481582 | 21,51 | Decreased |
| MT-binding proteins | Map1a_S2620 | -1,434040827 | 29,53 | Decreased |
| MT-binding proteins | Map1a_S2620 | -1,123912585 | 25,99 | Decreased |
| MT-binding proteins | Map1a_S2627 | -1,434040827 | 29,53 | Decreased |
| MT-binding proteins | Map1a_S2627 | -1,574629727 | 19,44 | Decreased |
| MT-binding proteins | Map1a_S2635 | -1,123912585 | 25,99 | Decreased |
| MT-binding proteins | Map1a_S2635 | -2,550832432 | 19,03 | Decreased |
| MT-binding proteins | Map1a_S2635 | -1,15822909 | 35,12 | Decreased |
| MT-binding proteins | Map1a_S2635 | -1,574629727 | 19,44 | Decreased |
| MT-binding proteins | Map1a_S2635 | -1,312791974 | 24,28 | Decreased |
| MT-binding proteins | Map1a_S499 | -1,282433972 | 44,96 | Decreased |
| MT-binding proteins | Map1a_S500 | -1,512127202 | 35,05 | Decreased |
| MT-binding proteins | Map1a_T2177 | -1,121785109 | 30,58 | Decreased |
| MT-binding proteins | Map1a_T2621 | -2,550832432 | 19,03 | Decreased |
| MT-binding proteins | Map1a_T2621 | -1,15822909 | 35,12 | Decreased |
| MT-binding proteins | Map1a_T504 | -1,017296749 | 19,7 | Decreased |
| MT-binding proteins | Map1a_T504 | -1,282433972 | 44,96 | Decreased |
| MT-binding proteins | Map1a_T504 | -1,512127202 | 35,05 | Decreased |
| MT-binding proteins | Map1b_S1201 | -1,403457526 | 21,85 | Decreased |
| MT-binding proteins | Map1b_S1252 | -1,210560074 | 27,57 | Decreased |
| MT-binding proteins | Map1b_S1257 | -1,210560074 | 27,57 | Decreased |
| MT-binding proteins | Map1b_S1257 | -2,310198796 | 28,73 | Decreased |
| MT-binding proteins | Map1b_S1257 | -1,166382431 | 18,3 | Decreased |
| MT-binding proteins | Map1b_S1369 | -1,612764349 | 19,93 | Decreased |
| MT-binding proteins | Map1b_S1369 | -1,014395436 | 18,61 | Decreased |
| MT-binding proteins | Map1b_S1371 | -1,190247994 | 17,97 | Decreased |
| MT-binding proteins | Map1b_S1618 | -1,377010082 | 24 | Decreased |
| MT-binding proteins | Map1b_S1683 | -1,139769281 | 16,63 | Decreased |
| MT-binding proteins | Map1b_S2264 | -1,645518969 | 33,59 | Decreased |
| MT-binding proteins | Map1b_S2264 | -1,881141438 | 18,67 | Decreased |
| MT-binding proteins | Map1b_S541 | -1,039559347 | 22,84 | Decreased |
| MT-binding proteins | Map1b_S541 | -1,111386433 | 20,05 | Decreased |
| MT-binding proteins | Map1b_S544 | -1,00174817 | 20,71 | Decreased |
| MT-binding proteins | Map1b_S744 | -Inf | 82,14 | Decreased |
| MT-binding proteins | Map1b_S744 | -4,765681999 | 35,35 | Decreased |
| MT-binding proteins | Map1b_S988 | -1,257061979 | 25,45 | Decreased |
| MT-binding proteins | Map1b_T1274 | -2,103232287 | 15,18 | Decreased |
| MT-binding proteins | Map1b_T527 | -2,636490163 | 28,1 | Decreased |
| MT-binding proteins | Map1b_T741 | -4,765681999 | 35,35 | Decreased |
| MT-binding proteins | Map2_S1597 | -1,485630935 | 25,27 | Decreased |
| MT-binding proteins | Map2_S1637 | -1,717360598 | 26,54 | Decreased |
| MT-binding proteins | Map2_S1656 | -2,707313558 | 15,02 | Decreased |
| MT-binding proteins | Map2_S825 | -1,283637282 | 15,77 | Decreased |
| MT-binding proteins | Map2_T1611 | -1,485630935 | 25,27 | Decreased |
| MT-binding proteins | Map2_T1611 | -1,184244151 | 44,09 | Decreased |
| MT-binding proteins | Map2_T1634 | -1,717360598 | 26,54 | Decreased |
| MT-binding proteins | Map2_T1652 | -2,707313558 | 15,02 | Decreased |
| MT-binding proteins | Map2_T1652 | -1,237739911 | 15,26 | Decreased |
| MT-binding proteins | Map2_T1659 | -2,707313558 | 15,02 | Decreased |
| MT-binding proteins | Map2_T736 | -2,454242513 | 13,93 | Decreased |
| MT-binding proteins | Map2_T738 | -2,454242513 | 13,93 | Decreased |
| MT-binding proteins | Map4_S668 | -4,264182106 | 30,78 | Decreased |
| MT-binding proteins | Map4_T659 | -4,264182106 | 30,78 | Decreased |
| MT-binding proteins | Map4_T848 | -1,13272271 | 24,49 | Decreased |
| MT-binding proteins | Map6_S747 | -2,054430661 | 17,38 | Decreased |
| MT-binding proteins | Map7_S231 | -1,77431129 | 19,66 | Decreased |
| MT-binding proteins | Map7d1_S512 | -1,228159575 | 21,21 | Decreased |
| MT-binding proteins | Map7d2_S184 | -1,442178291 | 27,26 | Decreased |
| MT-binding proteins | Map7d2_S243 | -1,858057376 | 30,65 | Decreased |
| MT-binding proteins | Map7d2_S293 | -1,762587806 | 15,54 | Decreased |
| MT-binding proteins | Mapt_S667 | -1,601504892 | 26,14 | Decreased |
| MT-binding proteins | Mapt_S727 | -1,775939978 | 16,33 | Decreased |
| MT-binding proteins | Mapt_S727 | -1,139724879 | 17,92 | Decreased |
| MT-binding proteins | Mapt_T523 | -1,236060973 | 14,74 | Decreased |
| MT-binding proteins | Mapt_T725 | -3,088021828 | 22,65 | Decreased |
| Tubulin-sequestering proteins | Stmn1_S16 | -1,669749992 | 28,69 | Decreased |
| Tubulin-sequestering proteins | Stmn1_S16 | -1,156909439 | 29,32 | Decreased |
| Tubulin-sequestering proteins | Stmn1_S25 | -1,669749992 | 28,69 | Decreased |
| Tubulin-sequestering proteins | Stmn1_S38 | -1,472515857 | 18,14 | Decreased |
| Tubulin-sequestering proteins | Stmn2_S50 | -1,272646833 | 18,61 | Decreased |
| Tubulin-sequestering proteins | Stmn3_S50 | -1,962442485 | 38,69 | Decreased |
| Tubulin-sequestering proteins | Stmn3_S53 | -1,962442485 | 38,69 | Decreased |
| Tubulin-sequestering proteins | Stmn3_S68 | -2,587565898 | 15,73 | Decreased |
| Tubulin-sequestering proteins | Stmn3_S73 | -2,587565898 | 15,73 | Decreased |
| Tubulin-sequestering proteins | Stmn3_S73 | -2,412794031 | 13,15 | Decreased |

^2^-Inf: Detected only in control
