# Supplementary material for: Redox signaling modulates axonal microtubule organization and induces a specific phosphorylation signature of microtubule-regulating proteins
Source: Redox Biol. 2025 Apr 3;83:103626. doi: 10.1016/j.redox.2025.103626 (PMC12019850; doi:10.1016/j.redox.2025.103626)
Supplement: Multimedia component 2 [file mmc2.docx]

**Supplementary Table 2:** Differentially altered phosphorylation sites of microtubule-regulating proteins in response to arsenite (based on individual phosphopeptides).

| **Category** | | **Gene Phosphosite** | **Fold Change [log₂]** | **P Value [-10lg]** | **Change direction** |
| --- | --- | --- | --- | --- | --- |
| End-binding proteins | Clasp1_S1209 | | 1,282523396 | 13,99 | Increased |
| End-binding proteins | Clasp1_S786 | | Inf^1^ | 57,82 | Increased |
| End-binding proteins | Clasp1_S792 | | Inf | 57,82 | Increased |
| End-binding proteins | Clasp2_S1021 | | 3,649455404 | 14,95 | Increased |
| End-binding proteins | Clasp2_S1273 | | Inf | 79,07 | Increased |
| End-binding proteins | Clasp2_S326 | | 1,047207957 | 14,25 | Increased |
| End-binding proteins | Clasp2_S329 | | 1,047207957 | 14,25 | Increased |
| End-binding proteins | Clasp2_S330 | | 1,034104626 | 13,39 | Increased |
| End-binding proteins | Clasp2_S333 | | 1,034104626 | 13,39 | Increased |
| End-binding proteins | Clasp2_S410 | | 1,418884793 | 15,64 | Increased |
| End-binding proteins | Clasp2_S413 | | 1,418884793 | 15,64 | Increased |
| End-binding proteins | Clasp2_S495 | | 2,044783635 | 29,93 | Increased |
| End-binding proteins | Clasp2_T1271 | | Inf | 79,07 | Increased |
| MT-binding proteins | Map1a_S1037 | | 1,798146779 | 23,93 | Increased |
| MT-binding proteins | Map1a_S1274 | | Inf | 54,27 | Increased |
| MT-binding proteins | Map1a_S1715 | | 2,742649513 | 31,65 | Increased |
| MT-binding proteins | Map1a_S1784 | | 4,239424503 | 15,04 | Increased |
| MT-binding proteins | Map1a_S1803 | | 2,147579033 | 18,62 | Increased |
| MT-binding proteins | Map1a_S499 | | 2,209962891 | 21,48 | Increased |
| MT-binding proteins | Map1a_S500 | | 2,209962891 | 21,48 | Increased |
| MT-binding proteins | Map1a_S842 | | 1,396763581 | 22,16 | Increased |
| MT-binding proteins | Map1a_T1712 | | 2,742649513 | 31,65 | Increased |
| MT-binding proteins | Map1a_T1800 | | 2,610928671 | 16,23 | Increased |
| MT-binding proteins | Map1a_T1800 | | 4,239424503 | 15,04 | Increased |
| MT-binding proteins | Map1a_T1801 | | 2,404471025 | 16,68 | Increased |
| MT-binding proteins | Map1a_T2177 | | 1,888881214 | 18,3 | Increased |
| MT-binding proteins | Map1b_S1369 | | 2,296099802 | 17,94 | Increased |
| MT-binding proteins | Map1b_S1389 | | 1,921628757 | 14,05 | Increased |
| MT-binding proteins | Map1b_S1393 | | 1,921628757 | 14,05 | Increased |
| MT-binding proteins | Map1b_S1401 | | 1,921628757 | 14,05 | Increased |
| MT-binding proteins | Map1b_S1513 | | 2,16240524 | 16,2 | Increased |
| MT-binding proteins | Map1b_S1637 | | 1,985589321 | 23,44 | Increased |
| MT-binding proteins | Map1b_S1639 | | 1,722167016 | 20,05 | Increased |
| MT-binding proteins | Map1b_S1646 | | 1,985589321 | 23,44 | Increased |
| MT-binding proteins | Map1b_S1646 | | 1,722167016 | 20,05 | Increased |
| MT-binding proteins | Map1b_S1765 | | 1,094144633 | 18,23 | Increased |
| MT-binding proteins | Map1b_S1772 | | 1,094144633 | 18,23 | Increased |
| MT-binding proteins | Map1b_S1812 | | 1,359319824 | 13,95 | Increased |
| MT-binding proteins | Map1b_S1812 | | 2,047792611 | 15,21 | Increased |
| MT-binding proteins | Map1b_S1812 | | 2,696990955 | 26,21 | Increased |
| MT-binding proteins | Map1b_S1827 | | 2,047792611 | 15,21 | Increased |
| MT-binding proteins | Map1b_S1828 | | 2,696990955 | 26,21 | Increased |
| MT-binding proteins | Map1b_S1847 | | 2,097829706 | 20,75 | Increased |
| MT-binding proteins | Map1b_S1915 | | 1,525299122 | 23,04 | Increased |
| MT-binding proteins | Map1b_S1981 | | 2,554906544 | 22,74 | Increased |
| MT-binding proteins | Map1b_S2015 | | Inf | 84,76 | Increased |
| MT-binding proteins | Map1b_S2051 | | 3,934913927 | 39,46 | Increased |
| MT-binding proteins | Map1b_S541 | | 1,477322865 | 26,77 | Increased |
| MT-binding proteins | Map1b_S544 | | 1,477322865 | 26,77 | Increased |
| MT-binding proteins | Map1b_S601 | | 2,237190086 | 15,95 | Increased |
| MT-binding proteins | Map1b_S601 | | Inf | 60,79 | Increased |
| MT-binding proteins | Map1b_S614 | | 2,237190086 | 15,95 | Increased |
| MT-binding proteins | Map1b_T1804 | | 2,150697049 | 31,19 | Increased |
| MT-binding proteins | Map1b_T1833 | | 2,150697049 | 31,19 | Increased |
| MT-binding proteins | Map1b_T2024 | | Inf | 50,77 | Increased |
| MT-binding proteins | Map1b_T2030 | | Inf | 50,77 | Increased |
| MT-binding proteins | Map1b_Y1823 | | 1,359319824 | 13,95 | Increased |
| MT-binding proteins | Map1s_S458 | | 3,528047933 | 33,45 | Increased |
| MT-binding proteins | Map1s_S462 | | 3,528047933 | 33,45 | Increased |
| MT-binding proteins | Map1s_S659 | | 1,686886887 | 22,11 | Increased |
| MT-binding proteins | Map1s_S659 | | 1,861627473 | 17,3 | Increased |
| MT-binding proteins | Map1s_S669 | | 1,686886887 | 22,11 | Increased |
| MT-binding proteins | Map1s_S669 | | 1,861627473 | 17,3 | Increased |
| MT-binding proteins | Map1s_S784 | | 1,212238909 | 13,94 | Increased |
| MT-binding proteins | Map2_S1068 | | 2,711441229 | 13,21 | Increased |
| MT-binding proteins | Map2_S1166 | | 1,285740858 | 13,61 | Increased |
| MT-binding proteins | Map2_S1177 | | 1,626702413 | 20,13 | Increased |
| MT-binding proteins | Map2_S285 | | 2,888599933 | 17,1 | Increased |
| MT-binding proteins | Map2_S285 | | 1,904970154 | 14,55 | Increased |
| MT-binding proteins | Map2_S728 | | Inf | 66,48 | Increased |
| MT-binding proteins | Map2_S728 | | Inf | 66,76 | Increased |
| MT-binding proteins | Map2_T1161 | | 1,685729856 | 18,17 | Increased |
| MT-binding proteins | Map2_T1165 | | 1,685729856 | 18,17 | Increased |
| MT-binding proteins | Map2_T1165 | | 1,285740858 | 13,61 | Increased |
| MT-binding proteins | Map2_T290 | | 2,888599933 | 17,1 | Increased |
| MT-binding proteins | Map2_T290 | | 1,904970154 | 14,55 | Increased |
| MT-binding proteins | Map2_T736 | | Inf | 66,48 | Increased |
| MT-binding proteins | Map2_T738 | | Inf | 66,76 | Increased |
| MT-binding proteins | Map2_T752 | | Inf | 66,76 | Increased |
| MT-binding proteins | Map2_T753 | | Inf | 66,48 | Increased |
| MT-binding proteins | Map7d1_S256 | | 2,222225821 | 17,68 | Increased |
| MT-binding proteins | Map7d2_S619 | | 1,791211298 | 24,85 | Increased |
| MT-binding proteins | Mapt_S239 | | Inf | 77,28 | Increased |
| MT-binding proteins | Mapt_S342 | | 2,920600252 | 15,82 | Increased |
| MT-binding proteins | Mapt_S374 | | 1,260292803 | 13,44 | Increased |
| MT-binding proteins | Mapt_S667 | | 1,833855252 | 13,79 | Increased |
| Tubulin-sequestering proteins | Stmn1_S25 | | 1,784736899 | 19,23 | Increased |
| Tubulin-sequestering proteins | Stmn2_S62 | | 2,809455846 | 13,28 | Increased |
| Tubulin-sequestering proteins | Stmn2_S62 | | 3,630260789 | 16,21 | Increased |
| Tubulin-sequestering proteins | Stmn3_S60 | | Inf | 52,24 | Increased |
| Tubulin-sequestering proteins | Stmn3_S62 | | Inf | 66,25 | Increased |
| Tubulin-sequestering proteins | Stmn3_S68 | | Inf | 52,24 | Increased |
| Tubulin-sequestering proteins | Stmn3_S68 | | Inf | 66,25 | Increased |
| Tubulin isoforms | Tuba4a_S48 | | Inf | 55,02 | Increased |
| Nucleators | Tubgcp6_S1095 | | Inf | 55,31 | Increased |

^1^Inf: Detected only with arsenite

| **Category** | **Gene Phosphosite** | **Fold Change [log₂]** | **P Value [-10lg]** | **Change direction** |
| --- | --- | --- | --- | --- |
| End-binding proteins | Clasp1_S246 | -Inf^2^ | 49,25 | Decreased |
| End-binding proteins | Clasp1_S551 | -2,143462141 | 17,81 | Decreased |
| End-binding proteins | Clasp1_S553 | -2,143462141 | 17,81 | Decreased |
| End-binding proteins | Clasp1_S553 | -1,764880347 | 13,76 | Decreased |
| End-binding proteins | Clasp1_S555 | -1,764880347 | 13,76 | Decreased |
| MT-binding proteins | Map1a_S1007 | -1,016095506 | 13,87 | Decreased |
| MT-binding proteins | Map1a_S1007 | -1,555351232 | 18,92 | Decreased |
| MT-binding proteins | Map1a_S1191 | -1,497029016 | 13,12 | Decreased |
| MT-binding proteins | Map1a_S1271 | -1,792497182 | 13,38 | Decreased |
| MT-binding proteins | Map1a_S889 | -2,207217312 | 26,37 | Decreased |
| MT-binding proteins | Map1a_S894 | -2,207217312 | 26,37 | Decreased |
| MT-binding proteins | Map1a_S907 | -2,207217312 | 26,37 | Decreased |
| MT-binding proteins | Map1a_S990 | -1,016095506 | 13,87 | Decreased |
| MT-binding proteins | Map1a_S990 | -1,828251905 | 21,07 | Decreased |
| MT-binding proteins | Map1a_S990 | -1,555351232 | 18,92 | Decreased |
| MT-binding proteins | Map1a_T1002 | -1,828251905 | 21,07 | Decreased |
| MT-binding proteins | Map1b_S1148 | -1,724883305 | 15,31 | Decreased |
| MT-binding proteins | Map1b_S1244 | -1,47130719 | 28,34 | Decreased |
| MT-binding proteins | Map1b_S1244 | -1,169576879 | 17,35 | Decreased |
| MT-binding proteins | Map1b_S1248 | -Inf | 56,9 | Decreased |
| MT-binding proteins | Map1b_S1248 | -1,47130719 | 28,34 | Decreased |
| MT-binding proteins | Map1b_S1252 | -1,169576879 | 17,35 | Decreased |
| MT-binding proteins | Map1b_S1254 | -Inf | 56,9 | Decreased |
| MT-binding proteins | Map1b_S1257 | -1,47130719 | 28,34 | Decreased |
| MT-binding proteins | Map1b_S1382 | -2,053797693 | 16,23 | Decreased |
| MT-binding proteins | Map1b_S1613 | -2,845818028 | 13,74 | Decreased |
| MT-binding proteins | Map1b_S825 | -1,412459787 | 15,28 | Decreased |
| MT-binding proteins | Map1b_T1141 | -1,724883305 | 15,31 | Decreased |
| MT-binding proteins | Map1b_T1262 | -Inf | 56,9 | Decreased |
| MT-binding proteins | Map2_S741 | -1,069870528 | 23,21 | Decreased |
| MT-binding proteins | Map4_S761 | -Inf | 74,95 | Decreased |
| MT-binding proteins | Map4_S763 | -Inf | 74,95 | Decreased |
| MT-binding proteins | Mapt_S204 | -1,028042518 | 13,61 | Decreased |
| MT-binding proteins | Mapt_S204 | -1,088042342 | 25,6 | Decreased |
| MT-binding proteins | Mapt_S221 | -1,088042342 | 25,6 | Decreased |
| MT-binding proteins | Mapt_S509 | -1,262042867 | 17,32 | Decreased |
| MT-binding proteins | Mapt_S510 | -1,262042867 | 17,32 | Decreased |
| MT-binding proteins | Mapt_S707 | -4,059580229 | 32,64 | Decreased |
| MT-binding proteins | Mapt_S715 | -4,059580229 | 32,64 | Decreased |
| MT-binding proteins | Mapt_T213 | -1,028042518 | 13,61 | Decreased |

^2^-Inf: Detected only in control
